# Lipid metabolic reprogramming mediated by circulating Nrg4 alleviates metabolic dysfunction-associated steatotic liver disease during the early recovery phase after sleeve gastrectomy

**DOI:** 10.1186/s12916-024-03377-0

**Published:** 2024-04-17

**Authors:** Chengcan Yang, Dongzi Zhu, Chaofan Liu, Wenyue Wang, Yining He, Bing Wang, Meiyi Li

**Affiliations:** 1 Department of General Surgery, Shanghai Ninth People’s Hospital, Shanghai Jiao Tong University School of Medicine, Shanghai, 200011 China; 2 Biostatistics Office of Clinical Research Unit, Shanghai Ninth People’s Hospital, Shanghai Jiao Tong University School of Medicine, Shanghai, 200011 China; 3 Fudan Zhangjiang Institute, Fudan University, Shanghai, 201203 China

**Keywords:** Metabolic dysfunction-associated steatotic liver disease, Sleeve gastrectomy, Early postoperative recovery phase, Lipid metabolic reprogramming, Neuregulin-4, Fatty acid oxidation

## Abstract

**Background:**

The metabolic benefits of bariatric surgery that contribute to the alleviation of metabolic dysfunction-associated steatotic liver disease (MASLD) have been reported. However, the processes and mechanisms underlying the contribution of lipid metabolic reprogramming after bariatric surgery to attenuating MASLD remain elusive.

**Methods:**

A case–control study was designed to evaluate the impact of three of the most common adipokines (Nrg4, leptin, and adiponectin) on hepatic steatosis in the early recovery phase following sleeve gastrectomy (SG). A series of rodent and cell line experiments were subsequently used to determine the role and mechanism of secreted adipokines following SG in the alleviation of MASLD.

**Results:**

In morbidly obese patients, an increase in circulating Nrg4 levels is associated with the alleviation of hepatic steatosis in the early recovery phase following SG before remarkable weight loss. The temporal parameters of the mice confirmed that an increase in circulating Nrg4 levels was initially stimulated by SG and contributed to the beneficial effect of SG on hepatic lipid deposition. Moreover, this occurred early following bariatric surgery. Mechanistically, gain- and loss-of-function studies in mice or cell lines revealed that circulating Nrg4 activates ErbB4, which could positively regulate fatty acid oxidation in hepatocytes to reduce intracellular lipid deposition.

**Conclusions:**

This study demonstrated that the rapid effect of SG on hepatic lipid metabolic reprogramming mediated by circulating Nrg4 alleviates MASLD.

**Supplementary Information:**

The online version contains supplementary material available at 10.1186/s12916-024-03377-0.

## Background

Metabolic dysfunction-associated steatotic liver disease (MASLD) [1], formerly known as nonalcoholic fatty liver disease (NAFLD), is a complex systemic disease that has challenged medical and health care systems, especially in light of its increasing incidence worldwide. Although promising medicines are being developed, metabolic and bariatric surgery (MBS) is still an efficacious therapeutic strategy for patients with both obesity and MASLD. Recently, an increasing number of studies (including our previous studies) have demonstrated that MBS has important weight loss-dependent and weight loss-independent mechanisms for the remission or even resolution of metabolic disorders (e.g., MASLD, type 2 diabetes), and multiple target organs (e.g., the stomach, intestine, liver, adipose tissue) could benefit from metabolic reprogramming following MBS [2–5]. However, the precise and comprehensive mechanism involved in attenuating MASLD after MBS is not well understood. Some pieces of evidence have shown that improvements in systemic glucose homeostasis and insulin sensitivity account for the rapid effect of MSB on the remission of hepatic steatosis [6, 7], but investigations of lipid alterations in the early postoperative recovery phase are still lacking.

Secreted adipokines are strongly related to many obesity-associated metabolic diseases [8]. Accordingly, they can be released into the blood circulatory system and bind to specific receptors on the surface of target cells, activating signal transduction and mediating cross-talk among tissues to maintain systemic metabolic homeostasis. Thus, due to the ongoing global epidemic of obesity, adipokines have gained increasing attention due to their potential for both diagnosis and therapy. Recent studies have shown that several adipokines (e.g., leptin and adiponectin) can be influenced by physiological changes in gut adaptation after MBS [9]. Moreover, neuregulin-4 (Nrg4) has been shown to be strongly correlated with metabolic disorders (e.g., MASLD, metabolic syndrome) [10–12]. All of these findings have led to the use of adipokines as potential therapeutic agents for diet-induced hepatic steatosis [13, 14].

Hence, we designed a case–control study to evaluate the impact of three of the most common adipokines (Nrg4, leptin, and adiponectin) on alleviating hepatic steatosis during the early recovery phase following sleeve gastrectomy (SG, one of the most popular surgical methods). The purpose of this study was to provide new insights into the intertissue lipid metabolic reprogramming that occurs during the early stage after the SG surgical procedure.

## Methods

### Patients and volunteers

Consecutive morbidly obese patients (BMI ≥ 30 kg/m^2^) [15, 16] with MASLD [17] who underwent laparoscopic SG as previously described [18, 19] were recruited for this study between June 2019 and June 2020 from Shanghai Ninth People’s Hospital, Shanghai Jiao Tong University School of Medicine. Consecutive healthy volunteers without obesity and MASLD were also included from March to August 2021 as the control group. The inclusion and exclusion criteria are described in Additional file 1: Fig. S1. Clinical data and serum samples were collected from obese patients before and after SG (i.e., 1 month following surgery according to the Chinese guidelines for surgical treatment of obesity and type 2 diabetes mellitus [20]) as well as from healthy volunteers. Liver steatosis was diagnosed by abdominal CT according to a liver/spleen ratio (LSR) < 1 [18, 19, 21, 22].

### Human serum adipokine analyses

Serum Nrg4, leptin, and adiponectin were measured by using an enzyme-linked immunosorbent assay (ELISA) according to the guidelines of the corresponding ELISA kits (Elabscience, China).

### Rodent models

All mice were housed in a room with a controlled temperature (21–23 °C) and a 12-h light/12-h dark cycle under specific pathogen-free (SPF) conditions. MASLD (15-week-old male C57BL/6 J) and control (15-week-old male C57BL/6 J) mice were obtained from GemPharmatech (Nanjing, China). The MASLD mice were continually fed a high-fat diet (60% of calories from fat; D12492; Research Diets) (HFD) for at least 8 weeks until they satisfied the following two criteria: (1) the average body weight of mice fed the HFD (60% kcal/fat; D12492; Research Diets) was more than 20% that of mice fed a normal-fat diet (NFD) [23, 24], and (2) the liver tissues were more than 33% hepatic steatosis according to the light microscopy results and in accordance with the MASLD Clinical Research Pathology Working Group Guidelines [25]. The MASLD and control mice were randomly divided into 4 groups: the MASLD-sham group (*n* = 10), the MASLD-SG group (*n* = 10), the control-sham group (*n* = 10), and the control-SG group (*n* = 10) (Fig. 2A). The surgical procedures for the mice were established as previously described [26]. All the mice were sacrificed in the second and fourth weeks after the surgical intervention.

Conventional Nrg4 knockout (KO) mice were commercially available (Cyagen Biotechnology, China) (Additional file 1: Fig. S2). The KO mice and their corresponding controls were fed the HFD diet (60% of calories from fat; D12492; Research Diets) until they satisfied the above two criteria for MASLD (i.e., between the ages of 8 and 20 weeks). The mice were randomly divided into five groups (WT-sham, WT-SG, WT-SG-inhibitor, KO-sham, and KO-SG) based on the results of genotypic identification (Fig. 3A). The KO-SG-inhibitor group was subjected to postoperative intraperitoneal injections of afatinib (HY-10261; MCE, USA) (20 mg/kg, 5 days per week) [27] after SG. All mice were sacrificed in the eighth week after the surgical intervention.

### Pathological liver examination

Hematoxylin and eosin (H&E) (Beyotime, China) and Oil Red O (Sigma–Aldrich, USA) staining of fresh liver tissue from mice were performed to analyze hepatic steatosis under confocal microscopy by independent pathologists.

### Mouse serum biochemical examination

The mouse liver function indicators (i.e., ALT, AST, GGT) and lipid profile parameters (i.e., TC, TG, HDL-c, LDL-c) were measured by using corresponding assay kits (Nanjing Jiancheng Bioengineering Institute, China). Serum Nrg4, leptin, adiponectin, and insulin levels were measured by using commercially available ELISA kits (Elabscience Biotechnology). These experiments were repeated at least three times independently.

### RNA-seq library construction and sequencing

Liver samples from the WT-SG, KO-sham, and KO-SG groups were randomly selected for transcriptome analysis. Total RNA was extracted from each sample using TRIzol Reagent (Life Technologies), after which the purity and integrity of the RNA were assessed via an RNA Nano 6000 Assay Kit with a Bioanalyzer 2100 system (Agilent Technologies, CA, USA). The sequencing libraries were generated with a NEBNext Ultra RNA Library Prep Kit (NEB, USA) following the manufacturer’s recommendations, and index codes were added to attribute the sequences to each sample. Clustering of the index-coded samples was performed on a cBot Cluster Generation System using the TruSeq PE Cluster Kit v3-cBot-HS (Illumina) according to the manufacturer’s instructions. After cluster generation, the library preparations were sequenced on an Illumina NovaSeq platform, and 150-bp paired-end reads were generated.

### RNA-seq data analysis

Raw data with low quality and adapter contaminants were removed, and the clean reads were aligned to the reference genome using HISAT2 v2.0.5. The gene expression levels were estimated by featureCounts v1.5.0-p3. Then, the differentially expressed genes (DEGs) were analyzed by DESeq2 in R (version 4.3), and the* p* value was adjusted by a false discovery rate < 0.05. To uncover the functional roles of Nrg4 in alleviating hepatic steatosis, we categorized the overlapping DEGs between the two groups, as shown in Fig. 4B. We used the clusterProfiler package in R (version 4.3) to perform KEGG pathway enrichment analysis of the Nrg4-associated genes.

### Cell culture

Both the AML-12 and WRL-68 cell lines were obtained from the American Type Culture Collection of the Chinese Academy of Sciences (ATCC, China) and cultured in DMEM/F12 supplemented with 10% fetal bovine serum (Gibco, USA) in humidified air containing 5% CO^2^ at 37 °C. Cells were treated with different concentrations of oleic acid (OA) (Sigma–Aldrich, USA) for 24 h, and an equal volume of 20% bovine serum albumin (BSA) was used as a control. Recombinant lentiviral vectors (Hanbio, China) were used to generate Nrg4-overexpressing cell lines. Individual colonies were selected by qRT–PCR and western blotting (Additional file 1: Figs. S5A and S5B).

### Cell proliferation via the CCK8 assay

In vitro assessment of cell proliferation was performed by using a Cell Counting Kit-8 (CCK-8) cell proliferation assay (Hanbio, China). The OA-treated cells were seeded into 96-well tissue culture plates at a density of 5 × 10^3^ cells per well. At each time point, 10 μl of CCK8 solution was added to each well. After incubation at 37 °C for 4 h, the absorbance at 450 nm was recorded using a microplate reader.

### Western blot

Total proteins were extracted from the cell and mouse tissue lysates using RIPA buffer supplemented with protease inhibitor (Beyotime, China) and quantitated using the BCA assay. Equal amounts of denatured proteins were separated on 10% polyacrylamide gels (EpiZyme, China) and then transferred to polyvinylidene fluoride (PVDF) membranes (Merck Millipore, USA). The membranes were washed with TBST (10 mM Tris [pH 8.0], 150 mM NaCl, and 0.05% Tween-20) and incubated with the appropriate primary antibody (1:1000) at 4 °C overnight, followed by incubation with an HRP-conjugated secondary antibody (1:5000) (Cell Signaling Technology, USA) at room temperature for 1 h. The labeled proteins were visualized using ECL reagents (Merck Millipore, USA).

### Statistical analysis

The data are expressed as the mean ± SD. Statistical analysis was performed using an unpaired two-sided Student’s *t* test for comparisons between groups and paired two-sided Student’s *t* test for comparisons between pre-SG and post-SG groups. We used simple linear regression to evaluate the associations between adipokines and the variables of interest. *P* values < 0.05 were considered to indicate statistical significance. The following data were obtained for the adipokines and variables of interest: Nrg4, leptin, adiponectin, BMI, waist circumference (WC), waist-to-height ratio (WHtR), LSR, TC, TG, HDL-c, LDL-c ALT, AST, GGT, fasting glucose, fasting insulin, and HOMA-IR. All the statistical analyses were carried out with GraphPad Prism 9 (GraphPad, USA) software.

## Results

### The alleviation of hepatic steatosis in bariatric surgery patients with SG occurred during the early postoperative recovery phase

**Fig. 1:**
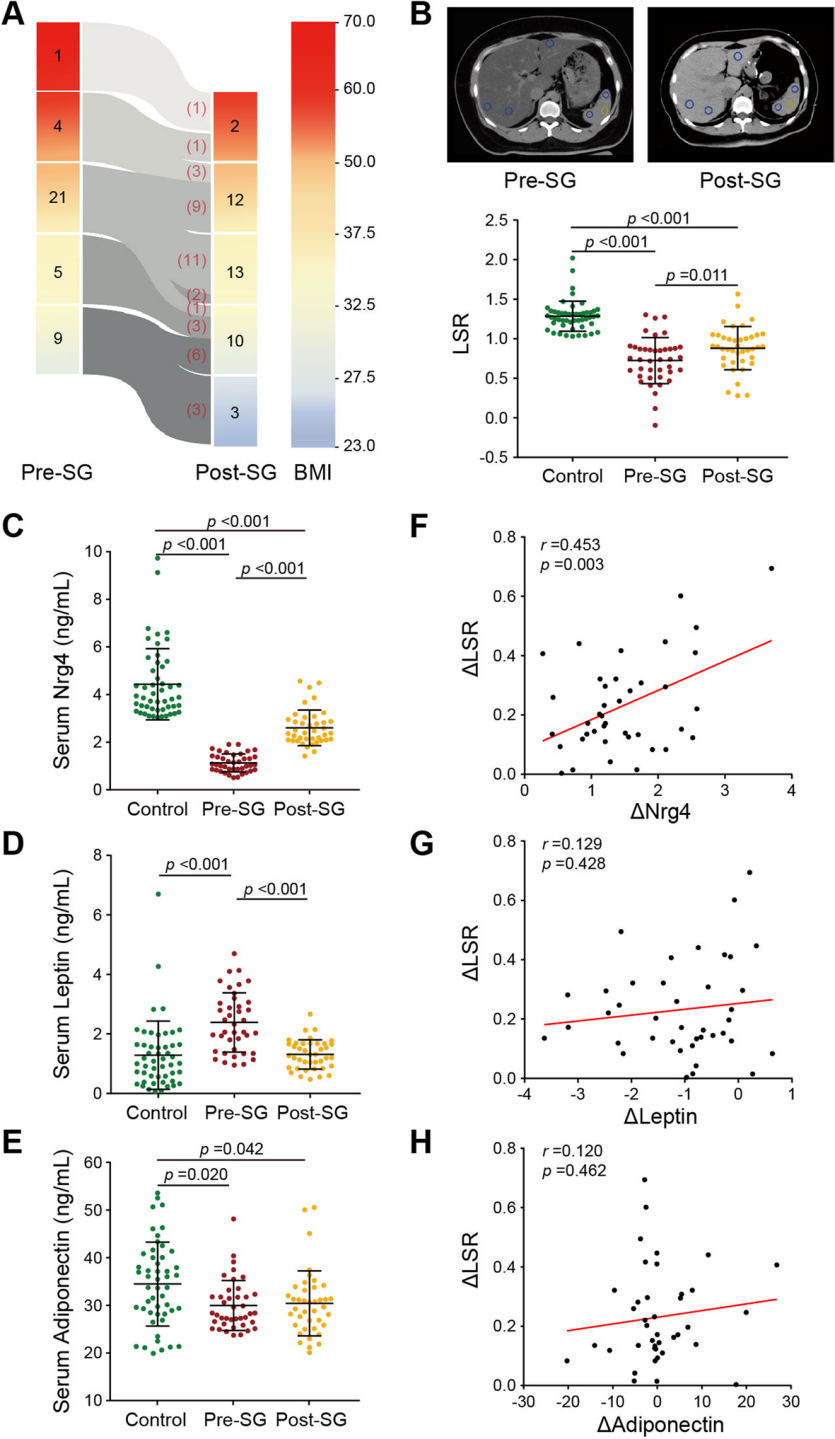
**A** Dynamics of BMI between the pre-SG group (*n* = 40) and post-SG group (*n* = 40). The color scale is shown on the right. **B** The image above shows the changes in the CT scan before and after SG. The image below shows the comparison of LSRs across the control, pre-SG, and post-SG groups. Profiles of the adipokines (Nrg4 (**C**), leptin (**D**), and adiponectin (**E**)) in the control, pre-SG, and post-SG groups and correlations between the adipokines and clinical parameters, including the LSR and BMI. The symbol Δ represents the post-SG group minus the pre-SG group. LSR, liver/spleen CT value ratio; BMI, body mass index. The data are presented as the means ± SDs. Each data point represents an individual patient

Forty consecutive obese patients with MASLD were recruited for liver and glycolipid metabolism observation in the early recovery phase after SG according to the inclusion and exclusion criteria (Additional file 1: Fig. S1). As shown in Table 1 and Additional file 1: Fig. S3, clinical characteristics, including liver CT scan, liver function, lipid profile, and glycometabolism, were collected from the patients before (i.e., pre-SG) and after (i.e., post-SG) laparoscopic SG. In addition, 50 healthy volunteers (i.e., controls) were enrolled in parallel. No severe complications were recorded among any of the patients. In this study, there were no significant differences in sex (*p* = 0.206), age (*p* = 0.187), or height (*p* = 0.066) between the patients and healthy volunteers (Table 1). Compared to the healthy volunteers, the obese patients before SG had significant hepatic steatosis (*p* < 0.001), hepatic dysfunction (i.e., ALT, AST, GGT; all *p* values < 0.001), dyslipidemia (i.e., TC, TG, HDL-c, LDL-c; all *p* values < 0.01 except TC), and dysglycemia (i.e., fasting glucose, fasting insulin, HOMA-IR; all *p* values ≤ 0.001) (Table 1 and Additional file 1: Fig. S3). A comparison of the clinical parameters of the obese patients before and after SG revealed nonsignificant reductions in weight (*p* = 0.494; Table 1) and BMI (*p* = 0.312; Table 1; Fig. 1A and Fig. S4A), and significant reductions were found in WC (*p* = 0.017; Table 1) and WHtR (*p* = 0.006; Table 1). Moreover, the liver/spleen CT value ratio (LSR) markedly increased (*p* = 0.011; Table 1), but no significant improvements were observed in liver function indicators (i.e., ALT, *p* = 1.000; AST, *p* = 0.299; GGT, *p* = 0.08; Table 1). However, the parameters of lipid and glucose metabolism markedly decreased (i.e., TC, *p* = 0.002; TG, *p* = 0.032; fasting glucose, *p* = 0.009; fasting insulin, *p* = 0.036; HOMA-IR, *p* = 0.013; Table 1 and Additional file 1: Fig. S3). Interestingly, these results suggested that the alleviation of hepatic steatosis as well as lipid and glucose metabolism occurred in the early recovery phase following SG without remarkable weight loss. Then, we analyzed the correlations between the metabolic parameters (including TC, TG, HDL-c, LDL-c, fasting glucose, fasting insulin, and HOMA-IR) and LSR (all *p* values > 0.05; Additional file 1: Fig. S5). There was no strong association between the improvement in serum glucose or lipid metabolism and the amelioration of hepatic steatosis in the early stage after bariatric surgery.

**Table 1.**
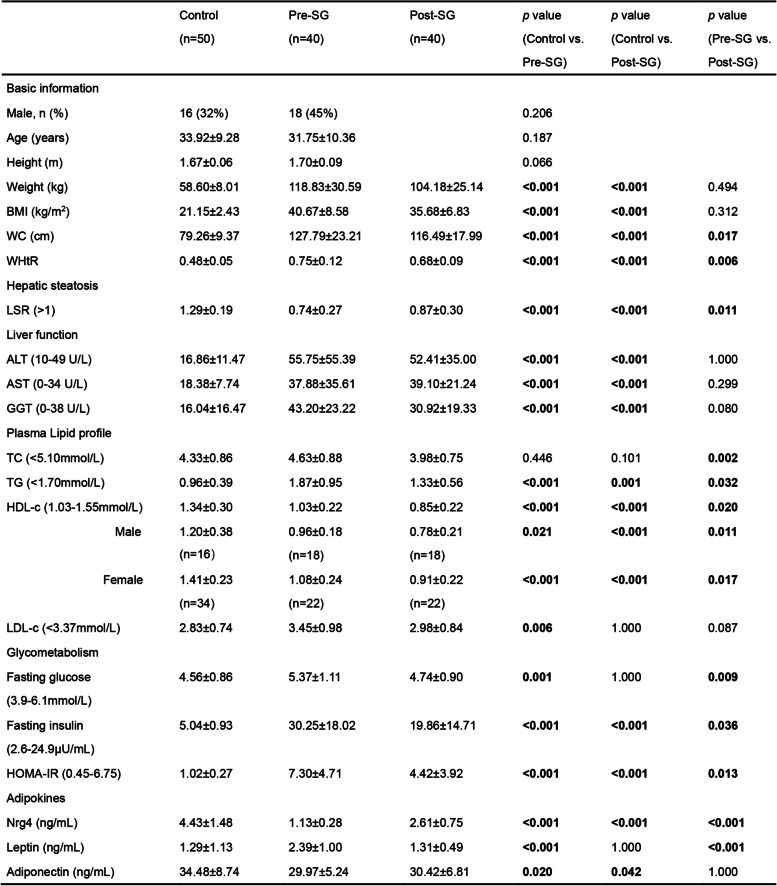
Clinical characteristics of the subjects included in this study

Bold values denote statistically significant *P* values. Control is a group of normal weight, non-operated health subjects. Pre-SG and Pre-SG imply patients with obesity before and the 1 month after sleeve gastrectomy, respectively. Categorical variables are presented as n (%), and continuous variables are presented as mean ± SD

*Abbreviations*: *BMI* body mass index, *WC* waist circumference, *WHtR* waist-to-height ratio, *LSR* liver/spleen CT value ratio, *ALT* alanine aminotransferase, *AST* aspartate aminotransferase, *GGT* gamma-glutamyl transferase, *TC* total cholesterol, *TG* triglyceride, *HDL-c* high-density lipoprotein cholesterol, *LDL-c* low-density lipoprotein cholesterol, *HOMA-IR* homeostasis model assessment insulin resistance, *Nrg4* neuregulin 4

### Hepatosteatosis alleviation is associated with an increase in circulating Nrg4 in the early recovery phase after SG

We next aimed to evaluate the effects of anatomical changes resulting from metabolic surgery on three typical secreted adipokines, Nrg4, leptin, and adiponectin, which are key mediators that participate in the intertissue regulation of lipid metabolism. Compared to those in the control individuals, the serum Nrg4 and adiponectin levels in the obese patients before SG were significantly lower, whereas the leptin level was significantly greater (all *p* values < 0.05; Table 1 and Fig. 1C to E). Comparison of the clinical parameters of the obese patients before and after SG revealed that serum Nrg4 levels increased significantly (*p* < 0.001, Table 1 and Fig. 1C), leptin levels decreased significantly (*p* < 0.001) and recovered to normal levels (Table 1 and Fig. 1D), and adiponectin levels were similar to those pre-SG (Table 1 and Fig. 1E). To test whether the alleviation of hepatic steatosis in the early stage after SG resulted from changes in the levels of these secreted adipokines, we subsequently analyzed the correlations between the three adipokines and the LSR (Fig. 1F to H). Only the changes in Nrg4 before and after SG were positively correlated with LSR improvement (*r* = 0.453, *p* = 0.003; Fig. 1F). We also performed correlation analysis of changes in BMI and each adipokine (i.e., Nrg4, leptin, and adiponectin) (Additional file 1: Fig. S4B). The results showed that the difference in adipokines was not related to changes in BMI after SG. Accordingly, these clinical results indicate that the alleviation of hepatic steatosis is strongly associated with the increase in circulating Nrg4 levels in the early recovery phase after bariatric surgery.

### Increase in the level of circulating Nrg4 in response to surgery in the early stage after surgery

**Fig. 2:**
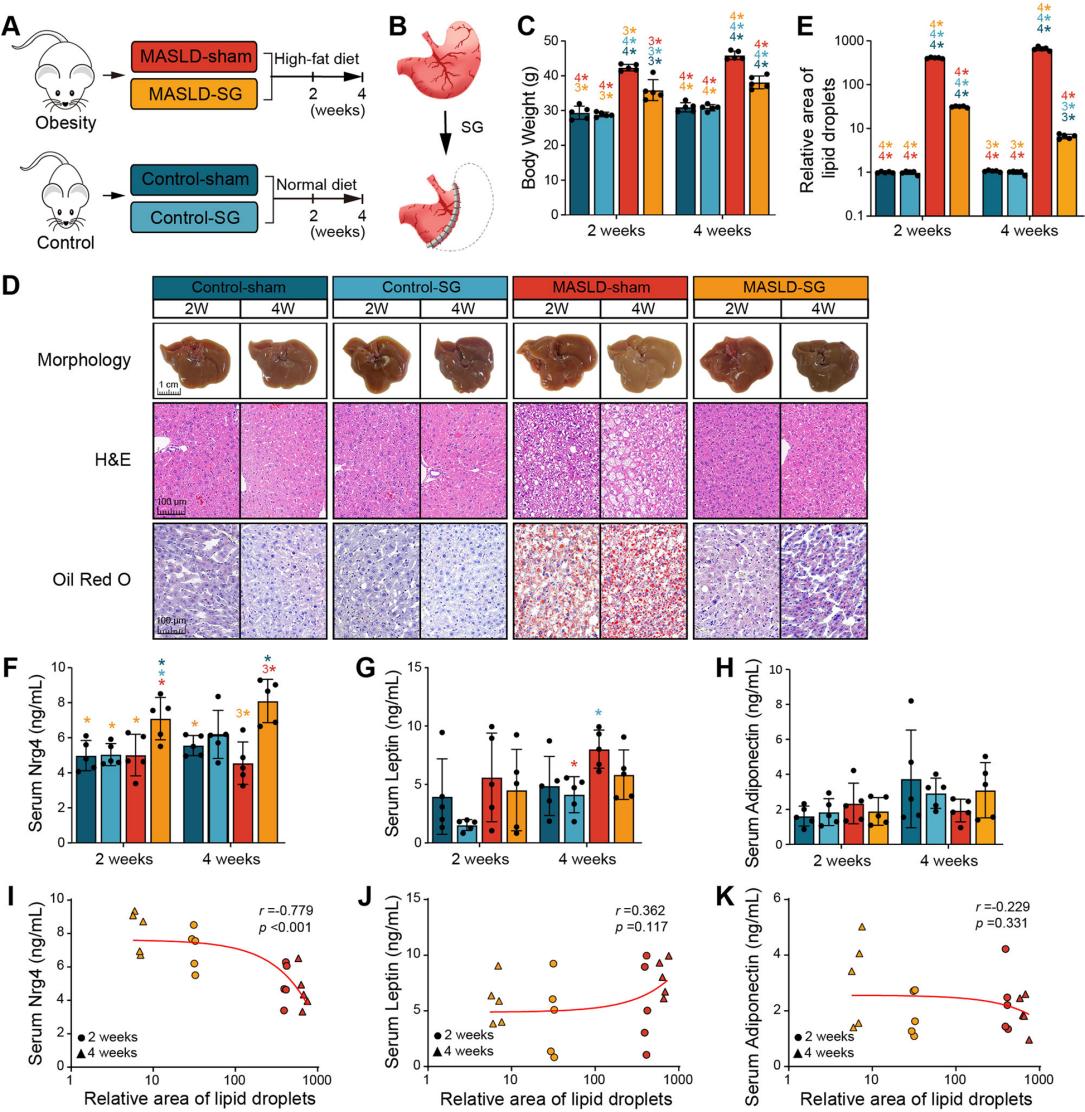
**A** Schematic diagram of rodent model construction and sample collection. **B** Surgical schematic diagram of sleeve gastrectomy (SG). **C** Body weight after SG in two groups of mice at 2 and 4 weeks post-surgery. **D** Representative images of morphology and H&E and Oil Red O staining of liver tissue sections from two groups of mice at 2 weeks and 4 weeks post-surgery. Scale bar, 1 cm or 100 μm. **E** Comparisons of the relative area of lipid droplets among the different groups of mice at 2 weeks and 4 weeks post-surgery. **F**–**H** Serum levels of the adipokines Nrg4 (**F**), leptin (**G**), and lipocalin (**H**) in 2 groups of mice. *n* = 5. * indicates *p* < 0.05, ^2^* indicates* p* < 0.01, ^3^* indicates* p* < 0.001, ^4^* indicates *p* < 0.0001. The color indicates the compared group. **I**–**K** Correlation analysis between adipokine levels and the relative area of lipid droplets

To investigate whether the increase in circulating Nrg4 is induced by SG surgery, we designed a series of rodent experiments to simulate the physiological changes in obese patients following bariatric surgery. As shown in Fig. 2A, a total of twenty 16-week-old weight-matched MASLD mice were randomly and evenly divided into two groups: the MASLD-sham group (*n* = 10) and the MASLD-SG group (*n* = 10). These two groups were continuously fed the HFD after undergoing either SG (Fig. 2B) or sham operation. Moreover, to determine the impact of surgical weight loss, 20 parallel control mice (normal weight) were also divided into two surgical groups corresponding to the MASLD groups but were fed a normal-fat diet (NFD) after surgical procedures. Five mice in each surgical group were sacrificed at 2 and 4 weeks after surgery for histological and biochemical (Additional file 1: Fig. S6) analyses.

Body weight was significantly greater in the two MASLD groups than in the two control groups at both the 2nd and 4th weeks after surgery (all *p* values < 0.001; Fig. 2C). For the control mice, no significant changes in body weight were observed between those subjected to SG and those subjected to sham operations. However, for the MASLD mice, the body weights of those subjected to SG were steadily lower than those of the sham-operated mice at both time points (both *p* values < 0.001; Fig. 2C). Then, we assessed hepatic steatosis and liver function among the groups via histological analyses. In terms of liver size and color, no significant changes were observed between the control-sham and control-SG groups at either time point, whereas the livers of the MASLD mice following SG almost recovered to the control level (Fig. 2D). H&E staining and Oil Red O-stained histopathology revealed fewer intracellular lipid droplets in the MASLD-SG group than in the MASLD-sham group (*p* < 0.0001; Fig. 2D and E). This alleviation of hepatic steatosis was more robust in the MASLD-SG mice than in the MASLD-sham mice. In particular, in the MASLD-SG group, the relative area of lipid droplets markedly decreased from 2 to 4 weeks after SG (Fig. 2E), which indicated that SG improved the remission of hepatic steatosis later in the postoperative phase after SG. Taken together, these results indicate that remission of hepatic steatosis occurred in the early stage following bariatric surgery.

The serum Nrg4 concentration was greater in the MASLD-SG group than in the other groups (all *p* values < 0.05) and gradually increased in both the control-SG and MASLD-SG groups from 2 to 4 weeks after surgery (Fig. 2F). This observation agreed with the observation of a significant increase in the serum Nrg4 concentration following SG in patients. There was almost no significant difference in the serum leptin or adiponectin concentrations among the groups (Fig. 2G and H). Additionally, we examined the correlations between these three adipokines and hepatic lipid deposition (Fig. 2I to K). A significant negative correlation was found only between Nrg4 and hepatic lipid deposition (*r* =  − 0.779, *p* < 0.001; Fig. 2I). These results support the strong association between an increase in circulating Nrg4 levels and bariatric surgery as well as an increase in Nrg4 levels in response to bariatric surgery beginning in the early stage.

### Nrg4 directly contributes to the beneficial effect of SG on the alleviation of lipid deposition

**Fig. 3:**
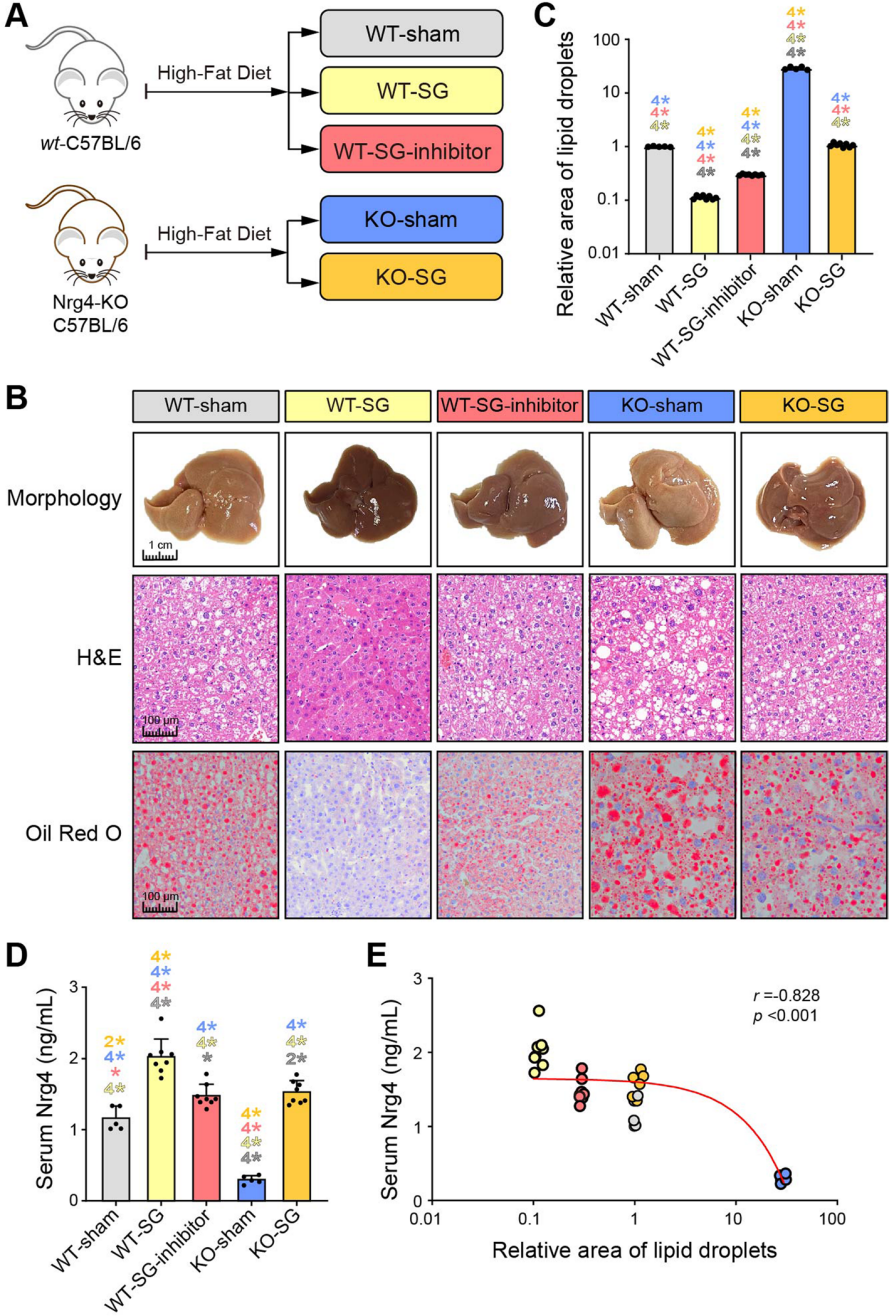
Gene deletion of Nrg4 increased susceptibility to MASLD and decreased the therapeutic effect of SG. **A** Schematic diagram of the rodent model construction. **B** Representative images of mouse liver tissue section morphology and H&E and Oil Red O staining. **C** Comparisons of the relative area of lipid droplets among the different groups of mice. **D** Comparisons of the serum Nrg4 concentration among different mouse models. * indicates *p* < 0.05, ^2^* indicates *p* < 0.01, ^3^* indicates *p* < 0.001, ^4^* indicates *p* < 0.0001. The color indicates the compared group. **E** Correlation analysis between Nrg4 and the relative area of lipid droplets in different mouse models

We further explored the functional role of circulating Nrg4 in this alleviation of hepatic steatosis after bariatric surgery. Both Nrg4-KO mice and wild-type (WT) mice were fed the HFD to promote the development of MASLD. Then, they were randomly divided into two groups and subjected to either SG or sham surgery (Fig. 3A). Because circulating Nrg4 was found to participate in the metabolic regulation of target cells by activating ErbB receptors (especially ErbB4) for signal transduction [13, 28], we randomly selected several WT-SG mice for treatment with afatinib (a pan-ErbB inhibitor) postoperatively (a WT-SG inhibitor). In terms of both size and color, the livers of the mice subjected to SG were better than those subjected to sham surgery (Fig. 3B). H&E and Oil Red O staining revealed remission of hepatic steatosis in WT-SG mice (Fig. 3B). The intracellular lipid droplet accumulation in the KO-SG group was similar to that in the WT-sham group (*p* > 0.05; Fig. 3B and C), which suggests that the loss of Nrg4 reduced the beneficial effect of SG on lipid deposition. However, the degree of lipid deposition in the WT-SG-inhibitor group was greater than that in the WT-SG group but lower than that in both the WT-sham and KO-SG groups (all *p* values < 0.0001; Fig. 3C), which suggests that circulating Nrg4 mainly depended on ErbB to reduce hepatic lipid deposition. Moreover, we observed that hepatic steatosis was much more severe in Nrg4-KO-sham mice than in the other groups (all *p* values < 0.0001; Fig. 3C), which indicates that Nrg4 protects against hepatic steatosis, as previously reported [13]. As expected, the serum Nrg4 concentration was significantly greater in the mice subjected to SG (including WT-SG, WT-SG-inhibitor, and Nrg4-KO-SG) than in those subjected to sham surgery (WT-sham and Nrg4-KO-sham) (all *p* values < 0.05; Fig. 3D). This result confirmed that bariatric surgery stimulated an increase in the circulating Nrg4 concentration. We subsequently examined the correlation between the Nrg4 concentration and hepatic lipid deposition and found a strongly negative correlation (*r* =  − 0.828, *p* < 0.001; Fig. 3E), similar to the abovementioned results (Fig. 2I). Taken together, these observations indicate that bariatric surgery, which depends on an increase in the circulating Nrg4 concentration, alleviates hepatic steatosis.

### Increased fatty acid oxidation induced by increases in Nrg4 following SG reduces lipid deposition

**Fig. 4:**
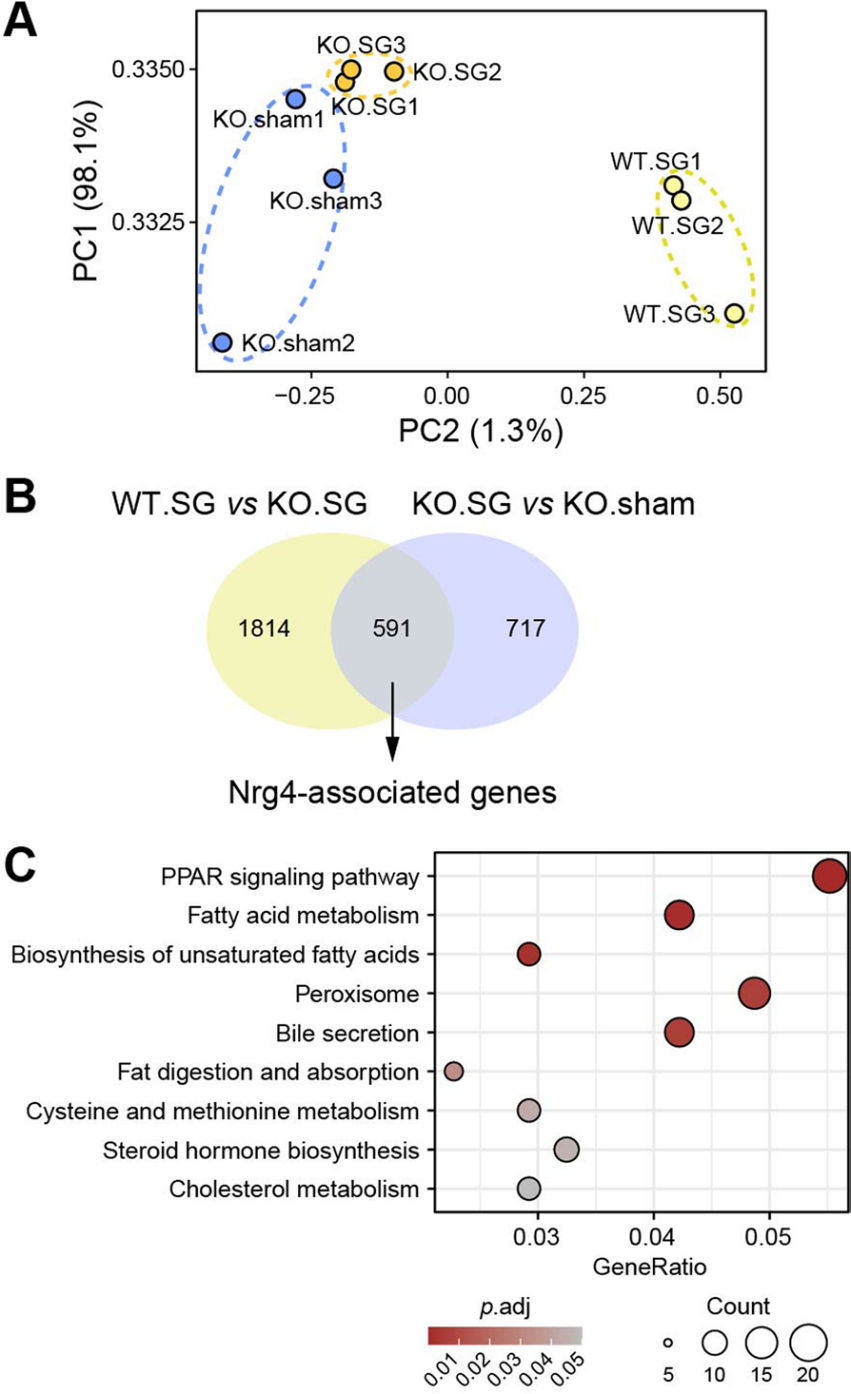
**A** PCA result of sample clustering based on DEGs. **B** Schematic diagram of the identification of Nrg4-associated genes. **C** Functional analysis of Nrg4-associated genes

Due to convincing evidence of a link between an increase in Nrg4 and the alleviation of hepatic steatosis, we questioned how circulating Nrg4 influenced hepatic lipid deposition. Thus, we performed transcriptional profiling of total RNA from liver tissues from WT-SG, KO-SG, and KO-sham mice to identify metabolic pathways regulated by Nrg4. A total of 3122 differentially expressed genes (DEGs) were identified by multiple comparisons with false discovery rate (FDR) adjustment (adjusted *p* < 0.05; Additional file 2: Table S1). After performing the principal component analysis (PCA) (Fig. 4A) based on the identified DEGs, we found that these samples were distinctly clustered into three independent groups representing different physiological states. The Nrg4-associated genes were further fractionated according to the functional roles of the DEGs (Fig. 4B and Additional file 2: Table S1). We subsequently performed functional analysis of the Nrg4-associated genes and identified a total of 9 enriched KEGG pathways (adjusted *p* < 0.05; Fig. 4C and Additional file 3: Table S2). The genes whose expression was altered by Nrg4 were involved mainly in lipid metabolism (e.g., fatty acid or cholesterol metabolism, biosynthesis of unsaturated fatty acids or steroid hormones). In particular, peroxisome proliferator-activated receptors (PPARs) are well known as fatty acid sensors and transcription factors that modulate lipid and glucose metabolism, especially fatty acid oxidation [29]. These results suggest that Nrg4 might play an important role in regulating fatty acid oxidation.

**Fig. 5:**
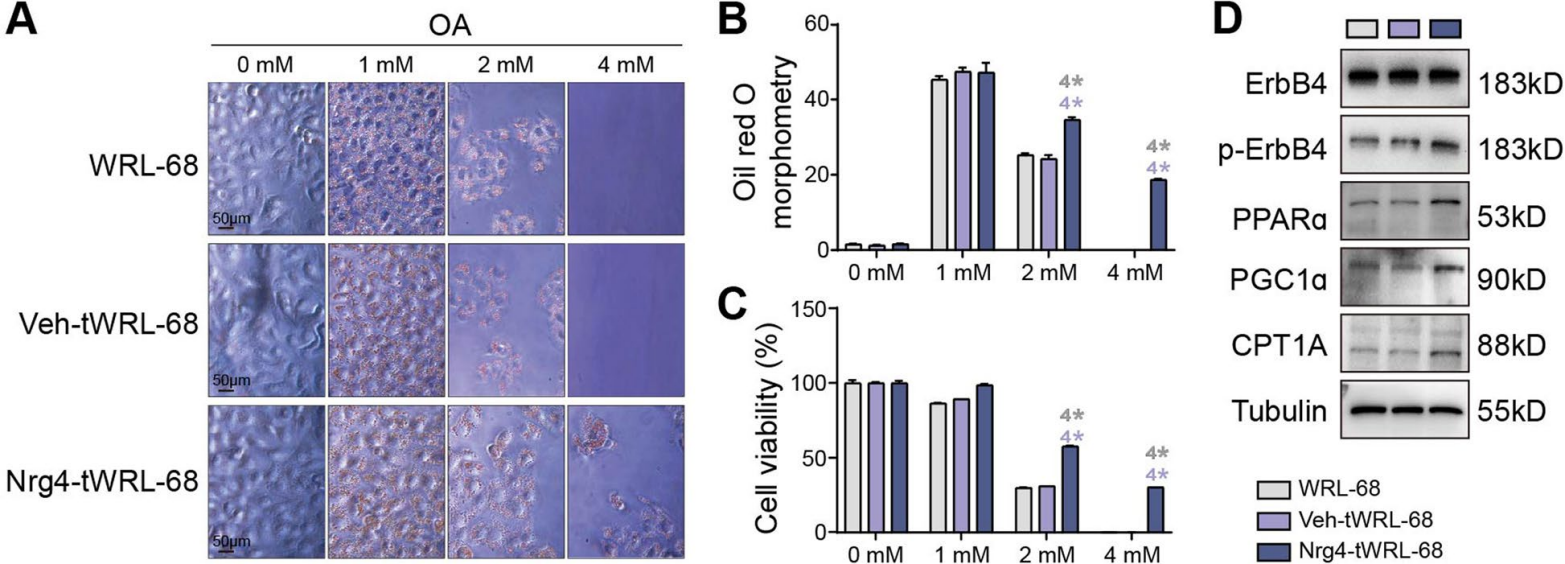
Characterization of the Nrg4 overexpression system in liver cell lines. **A** Oil Red O staining was used to analyze the metabolic status of control and Nrg4-overexpressing cells treated with different concentrations of OA. Scale bar, 50 μm. **B** Quantitative analysis of lipid droplets in panel **A**. **C** Cell viability of hepatocytes in panel A. **D** Western blot analysis of key proteins involved in fatty acid oxidation in control and Nrg4-overexpressing cell lines. * indicates *p* < 0.05, ^2^* indicates *p* < 0.01, ^3^* indicates* p* < 0.001, ^4^* indicates* p* < 0.0001. The color indicates the compared group

To clarify the functional role of Nrg4 in fatty acid oxidation, we performed gain-of-function studies to determine the effect of Nrg4 on lipid metabolism in hepatic cells. An Nrg4 overexpression system was generated in human WRL-68 (Additional file 1: Fig. S7A) and mouse AML-12 (Additional file 1: Fig. S7B) cell lines. We found that Nrg4 overexpression improved colony formation (Fig. 5A and B) and cell viability (Fig. 5C) at relatively high concentrations (> 2 mM) of OA. Then, we used western blotting to analyze the expression of several key molecules involved in fatty acid oxidation, including PPARa and PGC1a (the key transcription factors), CPT1A (the specific enzyme), and ErbB4 (the major receptor of Nrg4) (Fig. 5D). Nrg4 overexpression upregulated the expression of PPARa, PGC1a, and CPT1A but not ErbB4. However, we found that Nrg4 overexpression upregulated ErbB4 phosphorylation, which enhanced ErbB4 signal transduction (Fig. 5D). Additionally, we observed similar results in mouse hepatocytes (AML-12) (Additional file 1: Figs. S5C to S5F). Taken together, these results indicate that Nrg4 overexpression upregulated fatty acid oxidation.

**Fig. 6:**
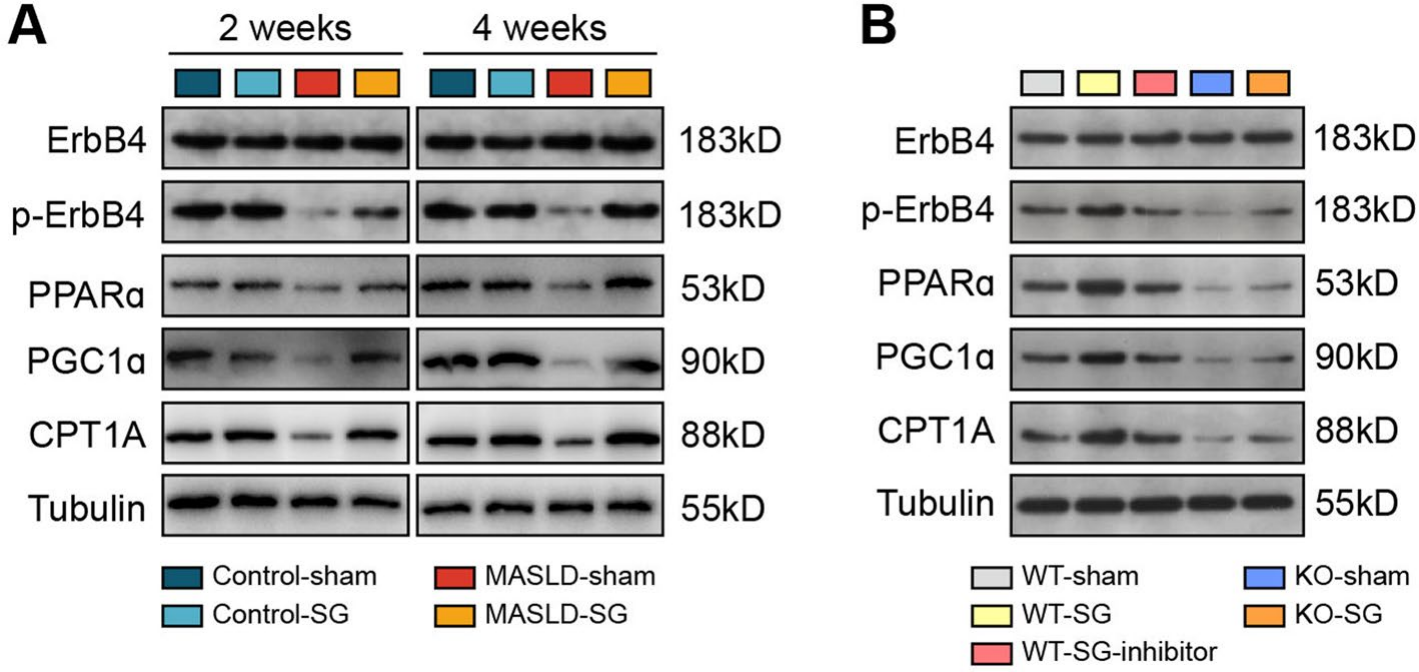
Western blot analysis of key proteins involved in fatty acid oxidation in the MASLD mouse models

To further validate the beneficial effect of SG on lipid metabolic reprogramming via an increase in circulating Nrg4 levels, we performed western blotting to analyze the expression of the above key molecules in the previous two mouse models. As shown in Fig. 6A, as expected, ErbB4 phosphorylation and the expression of PPARa, PGC1a, and CPT1A were significantly downregulated in the MASLD-sham group but seemed to return to normal in the MASLD-SG group. Moreover, ErbB4 phosphorylation and PPARa, PGC1a, and CPT1A expression were significantly upregulated in the WT-SG group but downregulated in the KO-sham and KO-SG groups (Fig. 6B). Taken together, these results verify that the beneficial effect of bariatric surgery on MASLD amelioration is partially attributed to hepatic lipid metabolic reprogramming via an increase in circulating Nrg4 levels.

## Discussion

With plentiful evidence-based data and long-term cohort studies emerging substantially over the past 20 years, the American Society for Metabolic and Bariatric Surgery (ASMBS) and the International Federation for the Surgery of Obesity and Metabolic Disorders (IFSO) issued a joint statement to update the indications for MBS [16, 30] earlier this year. MBS is regarded as an effective and long-lasting treatment for morbid obesity and its comorbidities (including insulin resistance and fatty liver disease) [31–35], but questions concerning the process and mechanism involved remain. In particular, it is not well understood whether the amelioration of obesity-related metabolic diseases benefits from weight loss or physiological changes after bariatric procedures. An increasing number of studies have gradually explored the dynamics of glucose metabolism after MBS (from early postoperative recovery to the weight loss maintenance phase) [6, 36]. Notably, glucose homeostasis improves early after bariatric surgery, before appreciable weight loss occurs [2, 3]. Additionally, an increase in multiple gut peptides (e.g., glucagon-like peptide 1, peptide YY) and bile acids as well as alteration of the gut microbiota following MBS have been reported to improve systemic glucose homeostasis and insulin sensitivity; these findings summarize the metabolic benefits of MBS for the remission of diabetes mellitus and MASLD independent of weight loss [6, 37]. In addition, simultaneous improvements in the blood lipid profile (e.g., triglyceride and cholesterol levels) were also observed beginning in the early postoperative period [38], which might indicate that improvements in systemic glucose homeostasis and insulin sensitivity cannot account entirely for the rapid effect on lipid metabolic reprogramming. Obviously, MASLD is a complex systemic disease, and its key pathogenic mechanisms could be related to dysfunction of lipid metabolism. Thus, the precise and comprehensive mechanisms underlying the attenuation of MASLD after MBS are not well understood; in particular, other potential contributors that directly and rapidly influence lipid metabolic reprogramming after bariatric procedures have not been identified.

Our study focused on the early postoperative recovery phase (i.e., at the 1-month follow-up after SG surgery in obese patients and before 8 weeks following SG surgery in mice) to investigate metabolic improvements following bariatric surgery. We observed that systemic glucose homeostasis and insulin sensitivity (as determined by fasting glucose, fasting insulin, and HOMA-IR values) improved in clinical patients, and lipid metabolic reprogramming (as indicated by TC, TG, and HDL-c levels) occurred rapidly after bariatric surgery. Moreover, liver function-associated parameters did not improve, whereas the LSR, which represents hepatic steatosis, markedly increased at the 1-month follow-up after SG. Similar observations were found in the comparison between obese mice subjected to SG and those subjected to a sham operation. These findings collectively suggest that the beneficial effect of bariatric surgery on metabolic reprogramming and the alleviation of hepatic steatosis began in the early postoperative recovery phase. Unexpectedly, there was no strong association between the improvement in glucose or lipid metabolism parameters from blood and the amelioration of hepatic steatosis in the early postoperative recovery phase.

We then investigated whether circulating adipokines might act as signal mediators to bridge physiological changes in gut adaptation and the rapid effect of intertissue lipid metabolic reprogramming after bariatric surgery. Here, we observed that morbidly obese patients exhibited an increase in circulating Nrg4 levels, which was associated with the amelioration of hepatic steatosis in the early recovery phase following SG before remarkable weight loss. Although a decrease in leptin levels and an increase in adiponectin levels following bariatric surgery were also supported by previous studies [9], they were not correlated with the variation in the LSR. By comparing obese mice subjected to SG and those subjected to sham surgery, we found that the increase in circulating Nrg4 levels was initially stimulated by SG and contributed to the ability of SG to reduce hepatic lipid deposition. Interestingly, an increase in circulating Nrg4 levels was not observed in normal mice subjected to SG, which is similar to GLP-1-associated systematic modulation of insulin toxicity [39]. Basically, GLP-1 enhances insulin release only when blood glucose is raised above the basal concentration, which might result from the plasticity of β-cell insulin secretory competence [40, 41]. Thus, GLP-1 agonists are considered dynamic triggers of weight loss without causing hypoglycemia.

**Fig. 7:**
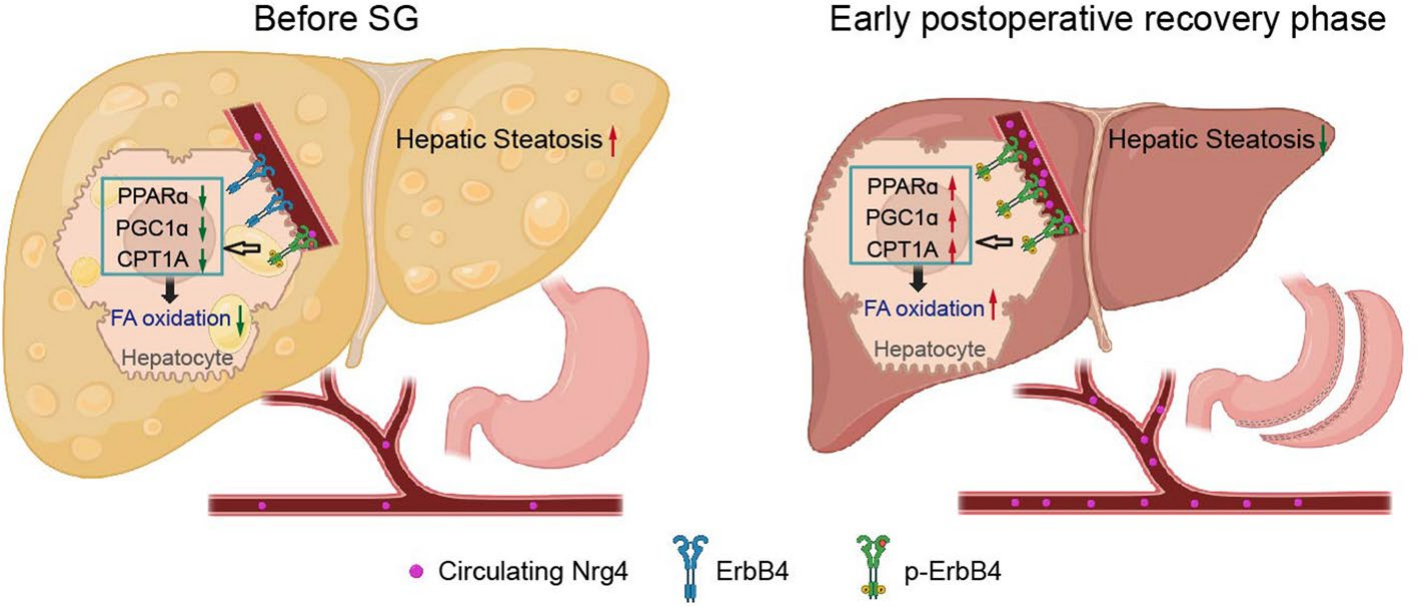
Schematic diagram of the rapid effect of SG on hepatic lipid metabolic reprogramming mediated by circulating Nrg4 for MASLD alleviation

Mechanistically, we explored how circulating Nrg4 mediates lipid metabolic reprogramming in the liver following bariatric surgery. By comparing a series of in vivo experiments, we found that circulating Nrg4 after SG activated ErbB4 on the surface of hepatocytes, which positively regulates fatty acid β-oxidation and negatively regulates de novo lipogenesis. This observation was confirmed in both mouse and human hepatic cell lines overexpressing Nrg4. It has been reported that Nrg4 can reach hepatocytes through the bloodstream, bind to ErbB4, and subsequently activate its downstream signaling pathway to regulate lipid metabolism in obese MASLD patients [13, 42, 43]. Accordingly, the alleviation of hepatic steatosis was partly attributed to the upregulation of fatty acid oxidation as well as the downregulation of de novo lipogenesis via Nrg4 after bariatric surgery (Fig. 7).

Although these findings suggest that SG-induced increases in circulating Nrg4 levels lead to the amelioration of MASLD, there are still several limitations to our study. First, an acknowledged limitation is that only a subset (three well-known adipokines whose concentration changes are strongly associated with bariatric surgery) of the larger circulating adipokine profile was examined. Second, clarification of the source of circulating Nrg4 following SG is lacking. Thus, because metabolic reprogramming following bariatric surgery involves complex and systemic interplay of multiple molecules and even tissues, the underlying mechanisms involved remain elusive. Future experiments to overcome the above limitations could encourage the consideration of Nrg4 as a therapeutic approach for MASLD in patients without the necessity for surgical intervention.

## Conclusions

Overall, the results of the present study demonstrated that the rapid effect of intertissue lipid metabolic reprogramming mediated by circulating Nrg4 after SG alleviates MASLD. Our findings provide new insights into the intertissue lipid metabolic reprogramming that occurs in the early recovery phase after bariatric surgery.

### Supplementary Information

**Additional file 1:** **Figure S1.** Screening flow chart of obese patients who underwent laparoscopic sleeve gastrectomy and healthy control of non-obese volunteers.**Figure S2.** Conventional Nrg4 knockout mice. **Figure S3.** Comparisons of clinical parameters among control, pre-SG, and post-SG groups. **Figure S4.** Comparison of BMI among control, pre-SG, and post-SG groups as well as correlations between adipokines and BMI. **Figure S5.** Correlations between metabolic parameters and LSR. **Figure S6.** Comparisons of metabolic parameters among different mice groups respectively at the 2nd and 4th postoperative weeks. **Figure S7.** Establishment and characterization of Nrg4 overexpression system in liver cell lines.

**Additional file 2:** **Table S1.** Differentially expressed genes.

**Additional file 3:** **Table S2. **Nrg4-assocaited KEGG pathways.

**Additional file 4.** Original blot images.

## Data Availability

The datasets generated and/or analyzed in the present study are available from the corresponding author upon reasonable request.

## Abbreviations

AC: Wild-type AML-12 cells without oleic acid treatment
AOA: Wild-type AML-12 cells treated with oleic acid
BMI: Body mass index
DEG: Differentially expressed gene
DIO: Diet-induced obesity
ErbB4: Erb-B2 receptor tyrosine kinase 4
HFD: High-fat diet
KO: Knockout
LSR: Liver/spleen CT value ratio
MASLD: Metabolic dysfunction-associated steatotic liver disease
NFD: Normal-fat diet
Nrg4: Neuregulin 4
NOA: Nrg4-overexpressing tAML-12 cells treated with oleic acid
SG: Sleeve gastrectomy
OA: Oleic acid
WC: Waist circumference
WHtR: Waist-to-height ratio
WT: Wild-type
