# Supplementary material for: Lipid metabolic reprogramming mediated by circulating Nrg4 alleviates metabolic dysfunction-associated steatotic liver disease during the early recovery phase after sleeve gastrectomy
Source: BMC Med. 2024 Apr 17;22:164. doi: 10.1186/s12916-024-03377-0 (PMC11025198; doi:10.1186/s12916-024-03377-0)
Supplement: Supplementary file 1 — Additional file 1: Figure S1. Screening flow chart of obese patients who underwent laparoscopic sleeve gastrectomy and healthy control of non-obese volunteers.Figure S2. Conventional Nrg4 knockout mice. Figure S3. Comparisons of clinical parameters among control, pre-SG, and post-SG groups. Figure S4. Comparison of BMI among control, pre-SG, and post-SG groups as well as correlations between adipokines and BMI. Figure S5. Correlations between metabolic parameters and LSR. Figure S6. Comparisons of metabolic parameters among different mice groups respectively at the 2nd and 4th postoperative weeks. Figure S7. Establishment and characterization of Nrg4 overexpression system in liver cell lines. [file 12916_2024_3377_MOESM1_ESM.docx]

**Supplementary Information**

**Lipid metabolic reprogramming mediated by circulating Nrg4 alleviates metabolic dysfunction-associated steatotic liver disease during the early recovery phase after sleeve gastrectomy**

- **Supplementary figures**

Figure S1: Screening flow chart of obese patients who underwent laparoscopic sleeve gastrectomy and healthy control of non-obese volunteers.

Figure S2: Conventional Nrg4 knockout mice.

Figure S3: Comparisons of clinical parameters among control, pre-SG, and post-SG groups.

Figure S4: Comparison of BMI among control, pre-SG, and post-SG groups as well as correlations between adipokines and BMI.

Figure S5: Correlations between metabolic parameters and LSR.

Figure S6: Comparisons of metabolic parameters among different mice groups respectively at the 2nd and 4th postoperative weeks.

Figure S7: Establishment and characterization of Nrg4 overexpression system in liver cell lines.

| **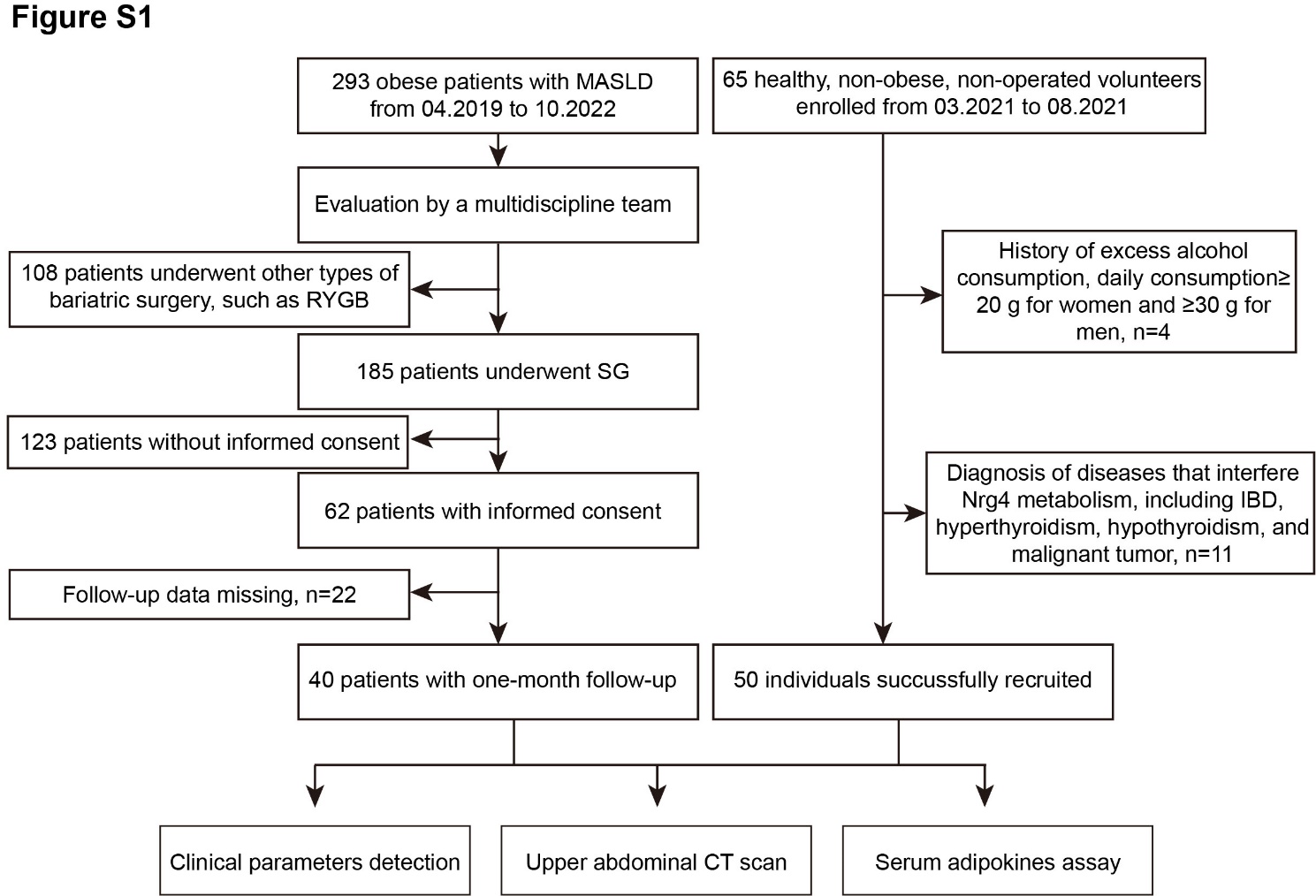** |
| --- |

**Figure S1. Screening flow chart of obese patients who underwent laparoscopic sleeve gastrectomy and healthy control of non-obese volunteers.** Inclusion criteria for patients: (1) Patients underwent SG, (2) Age between 18 and 60 years, (3) Liver/spleen HU ration (LSR) <1.0 in CT scan. Exclusion criteria for patients: (1) History of excess alcohol consumption (>21 standard drinks per week for men and >14 for women over 2 years, a standard alcoholic drink is any drink that contains about 14 g of pure alcohol); (2) Diagnosis of a liver disease that results in fatty liver, including viral hepatitis, autoimmune hepatitis, primary biliary cirrhosis, total parenteral nutrition, drug-induced liver disease, hemochromatosis, biliary obstruction, and Wilson’s disease; (3) Diagnosis of diseases that interfere Nrg4 metabolism, including inflammatory bowel disease, hyperthyroidism, hypothyroidism, and malignant tumor, and oral leukoplakia and oral lichen planus; (4) Without follow-up data 1 month after surgery. Inclusion criteria for volunteer group: (1) Age between 18 and 60 years, (2) BMI <25 kg/m^2^, (3) Undergoing CT scan and LSR should ≥1. Exclusion criteria for volunteers: (1) History of excess alcohol consumption (>21 standard drinks per week for men and >14 for women over 2 years, a standard alcoholic drink is any drink that contains about 14 g of pure alcohol); (2) Diagnosis of diseases that interfere Nrg4 metabolism, including inflammatory bowel disease, hyperthyroidism, hypothyroidism, and malignant tumor, and oral leukoplakia and oral lichen planus.

| ^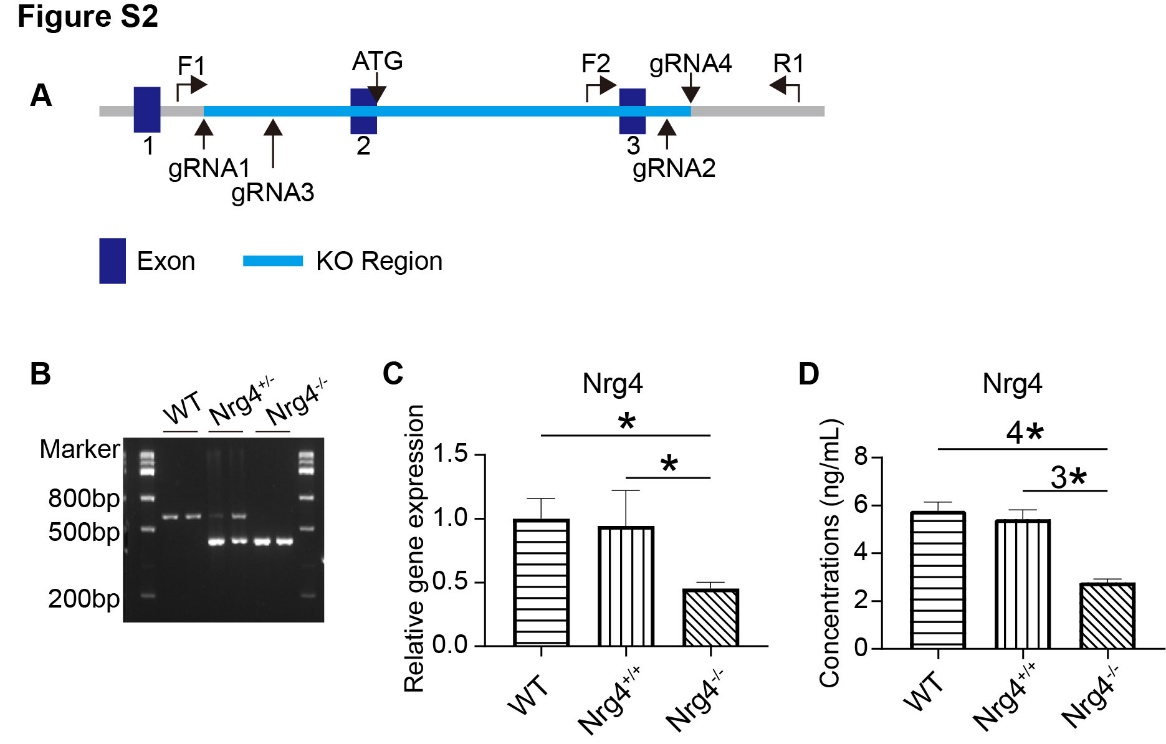^ |
| --- |

**Figure S2. Conventional Nrg4 knockout mice.** (A) Knock-out mice genotyping strategy. (B) Representative results of PCR screening. PCR primers 1 (annealing temperature 60.0 °C), F1: 5’-CTTGCAATCCCAATGTAAGGCTAAA-3’, R1: 5’-TTTGGAGGATGAACTATGATGTCCT-3’, product size: 420bp. PCR primers 2, F2: 5’-TATTTCGTGTGCATGTGTGTTGTAG-3’, R1: 5’-TTTGGAGGATGAACTATGATGTCCT-3’, Product size: 588 bp. Homozygotes: one band with 420 bp, Heterozygotes: two bands with 420 bp and 588 bp. Wildtype allele: one band with 588bp. (C) Comparison of expression levels of Nrg4 in mouse liver. (D) Comparison of concentration of Nrg4 in mouse serum. * indicates *p*<<0.05, ^2^* indicates *p*<0.01, ^3^* indicates *p*<0.001, ^4^* indicates *p*<0.0001.

| 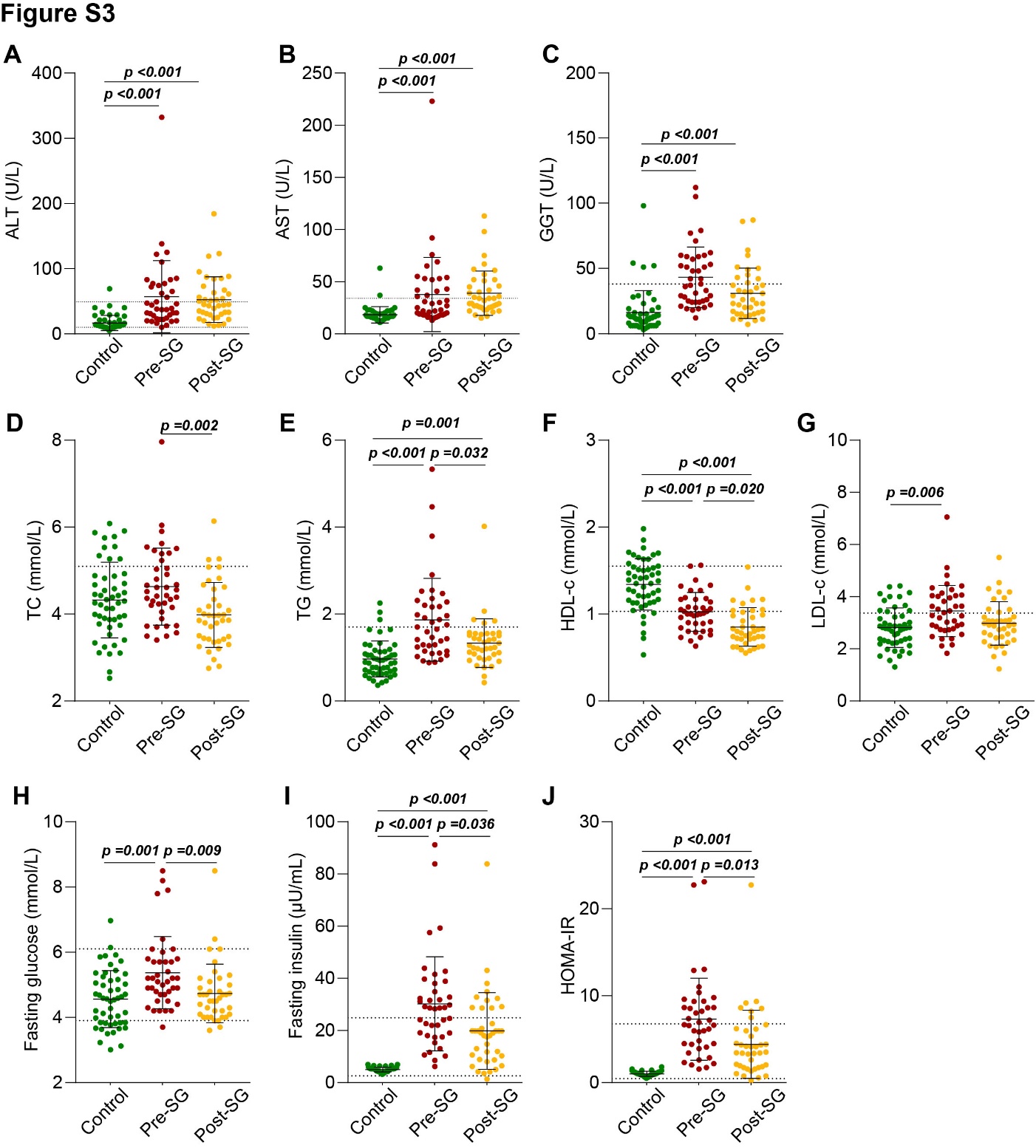 |
| --- |

**Figure S3. Comparisons of clinical parameters among control, pre-SG, and post-SG groups.** (A) ALT, alanine aminotransferase. (B) AST, aspartate aminotransferase. (C) GGT, gamma-glutamyl transferase. (D) TC, total cholesterol. (E) TG, triglyceride. (F) HDL-c, high-density lipoprotein cholesterol. (G) LDL-c, low-density lipoprotein cholesterol. (H) Fasting glucose. (I) Fasting insulin. (J) HOMA-IR, homeostasis model assessment insulin resistance. The dotted line represents the range of normal values. Data represented in mean ± SD. Each data point represents an individual patient. Related to Table 1.

| 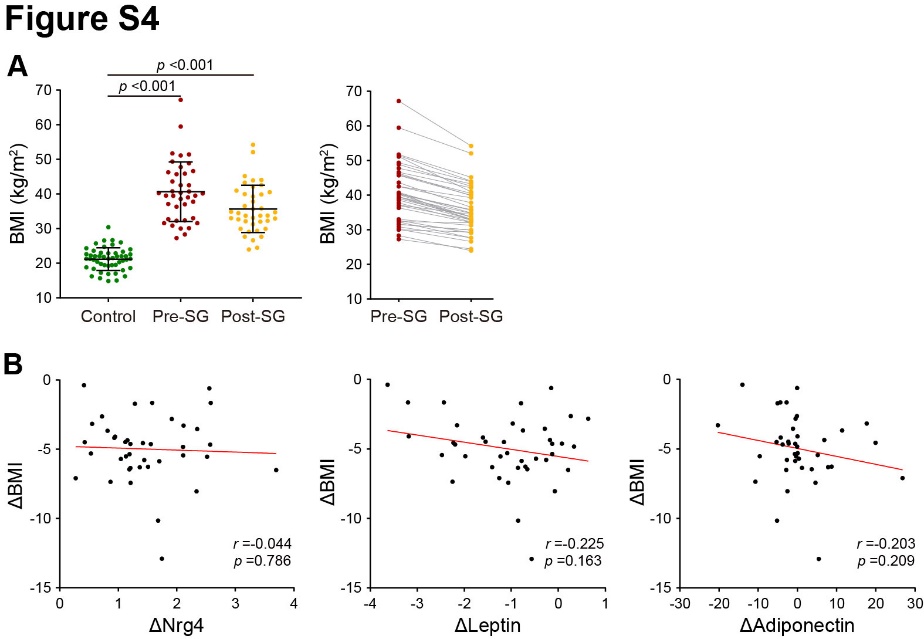 |
| --- |

**Figure S4.** (A) Comparison of BMI among control, pre-SG, and post-SG groups. (B) Correlations between adipokines and BMI. The symbol Δ represents post-SG group minus pre-SG group. Related to Table 1.

| 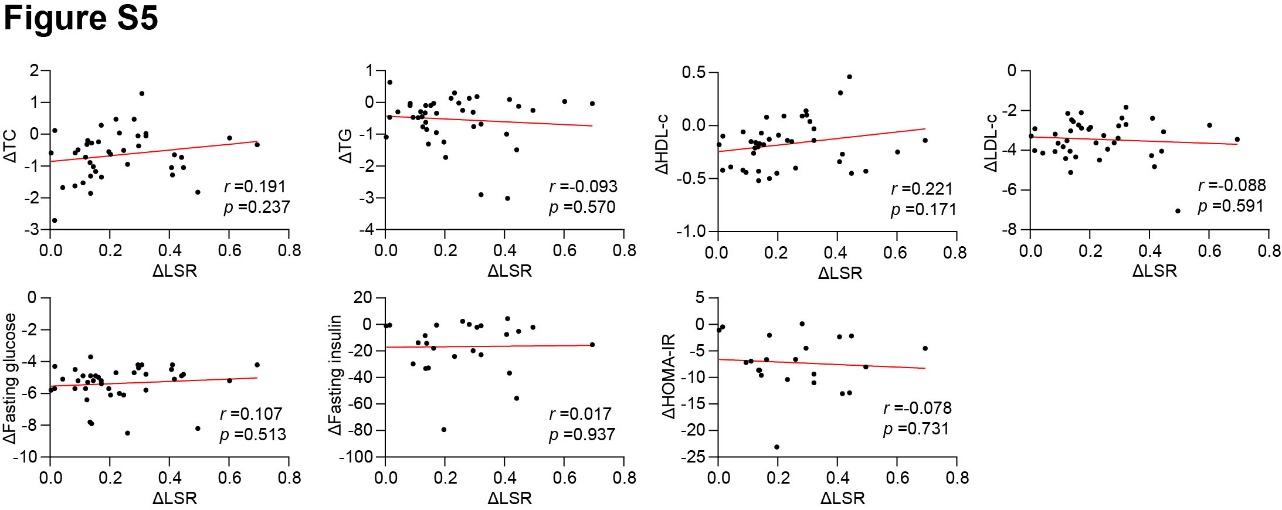 |
| --- |

**Figure S5. Correlations between metabolic parameters and LSR.** The symbol Δ represents post-SG group minus pre-SG group. LSR, liver/spleen CT value ratio; TC, total cholesterol; TG, triglyceride; HDL-c, high-density lipoprotein cholesterol; LDL-c, low-density lipoprotein cholesterol; HOMA-IR, homeostasis model assessment insulin resistance. Related to Table 1.

| 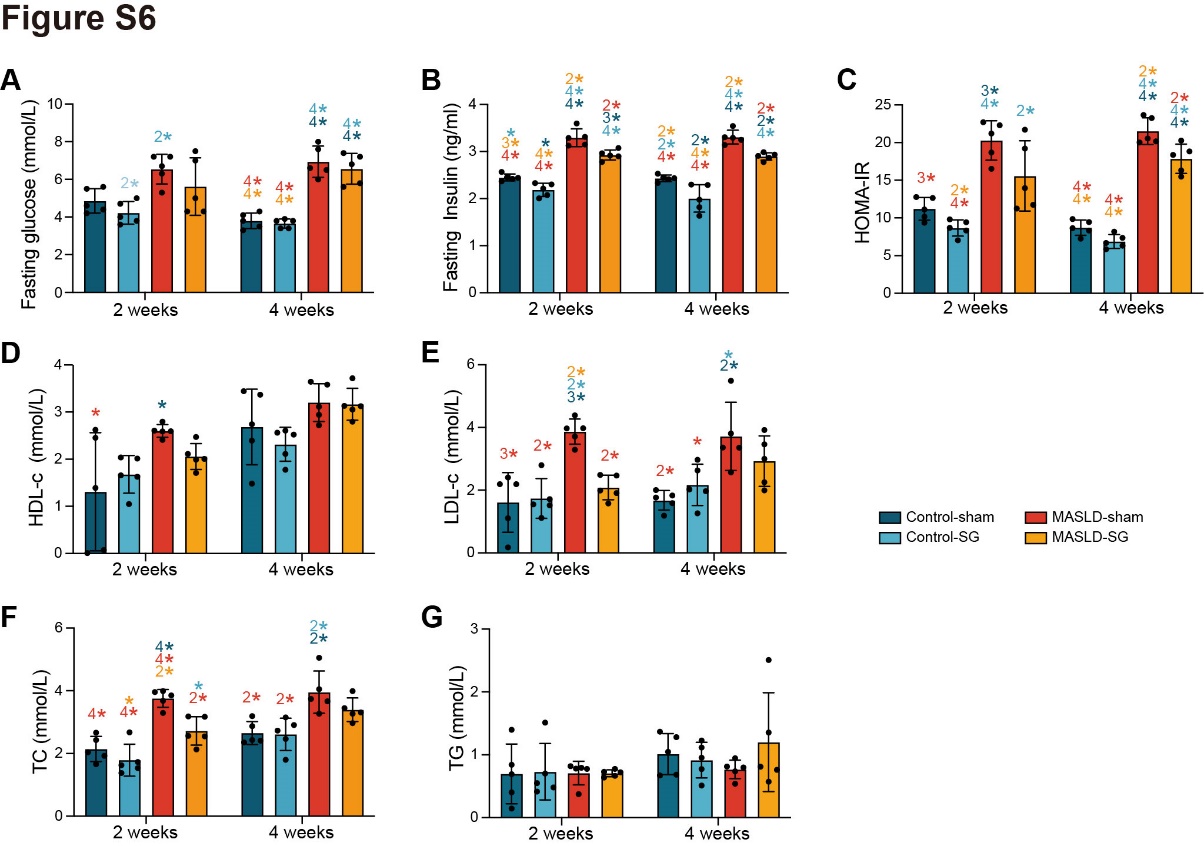 |
| --- |

**Figure S6. Comparisons of metabolic parameters among different mice groups at the 2nd and 4th postoperative weeks respectively.** (A-C) Serum levels of parameters related to glucose metabolism, including fasting glucose (A), fasting insulin (B), and HOMA-IR (C). (D-G) Serum levels of parameters related to lipid metabolism, including HDL-c (D), LDL-c (E), TC (F) and TG (G). * indicates *p*<0.05; ^2^* indicates *p*<0.01; ^3^* indicates *p*<0.001; ^4^* indicates *p*<0.0001. The color represents the compared group.

| 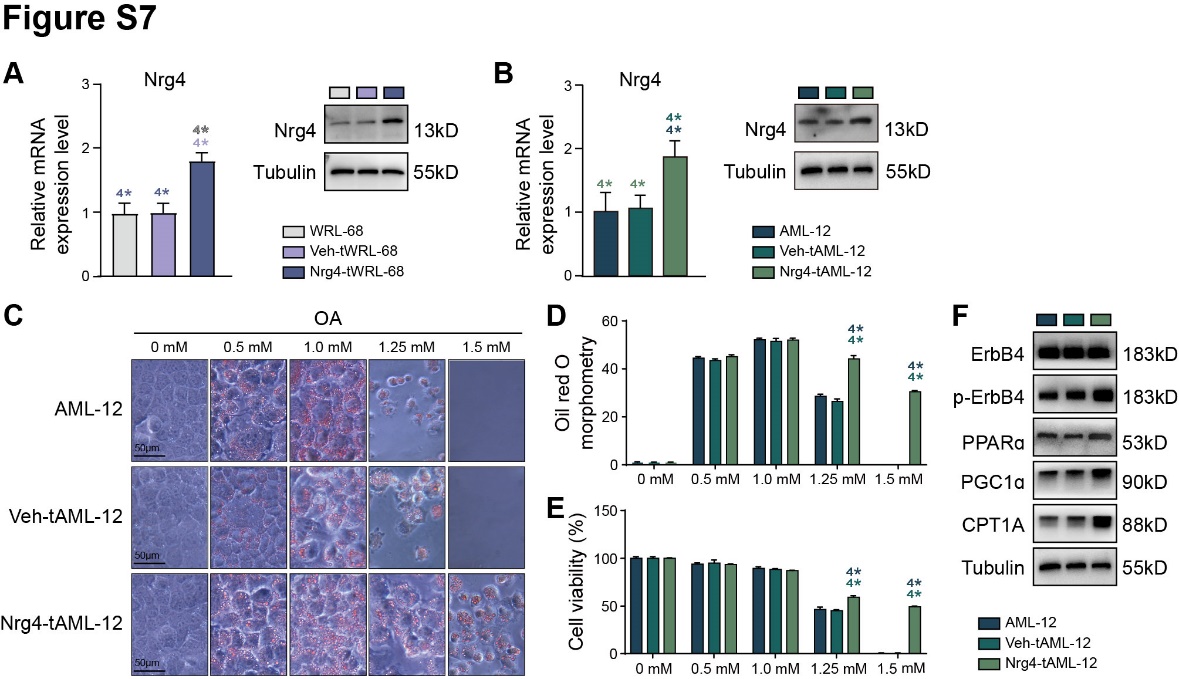 |
| --- |

**Figure S7. Establishment and characterization of Nrg4 overexpression system in liver cell lines.** (A-B) qPCR and western blot analyzed Nrg4 expression in human WRL-68 (A) and mouse AML-12 cell lines (B) with different treatments. (C) Oil red O staining analyzed metabolic status in control and Nrg4 overexpressed cell lines under different concentrations of OA. Scale bar, 50μm. (D) Quantitative analysis of lipid droplets in panel C. (E) Cell viability of hepatocytes in panel C. (F) Western blot analyzed key proteins of Nrg4-associated lipid metabolism in control and Nrg4 overexpressed cell lines. * represents *p*<0.05; ^2^* represents *p*<0.01; ^3^* represents *p*<0.001; ^4^* represents *p*<0.0001. The color represents the compared group.
