# Supplementary material for: Lipid metabolic reprogramming mediated by circulating Nrg4 alleviates metabolic dysfunction-associated steatotic liver disease during the early recovery phase after sleeve gastrectomy
Source: BMC Med. 2024 Apr 17;22:164. doi: 10.1186/s12916-024-03377-0 (PMC11025198; doi:10.1186/s12916-024-03377-0)
Supplement: Supplementary file 4 — Additional file 4. Original blot images. [file 12916_2024_3377_MOESM4_ESM.docx]

**Figure5. Original blot images**


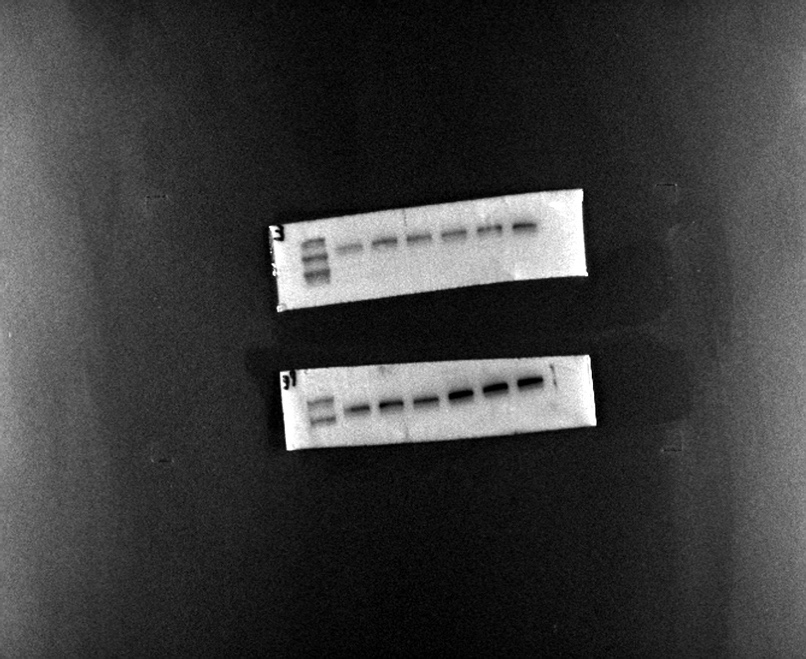

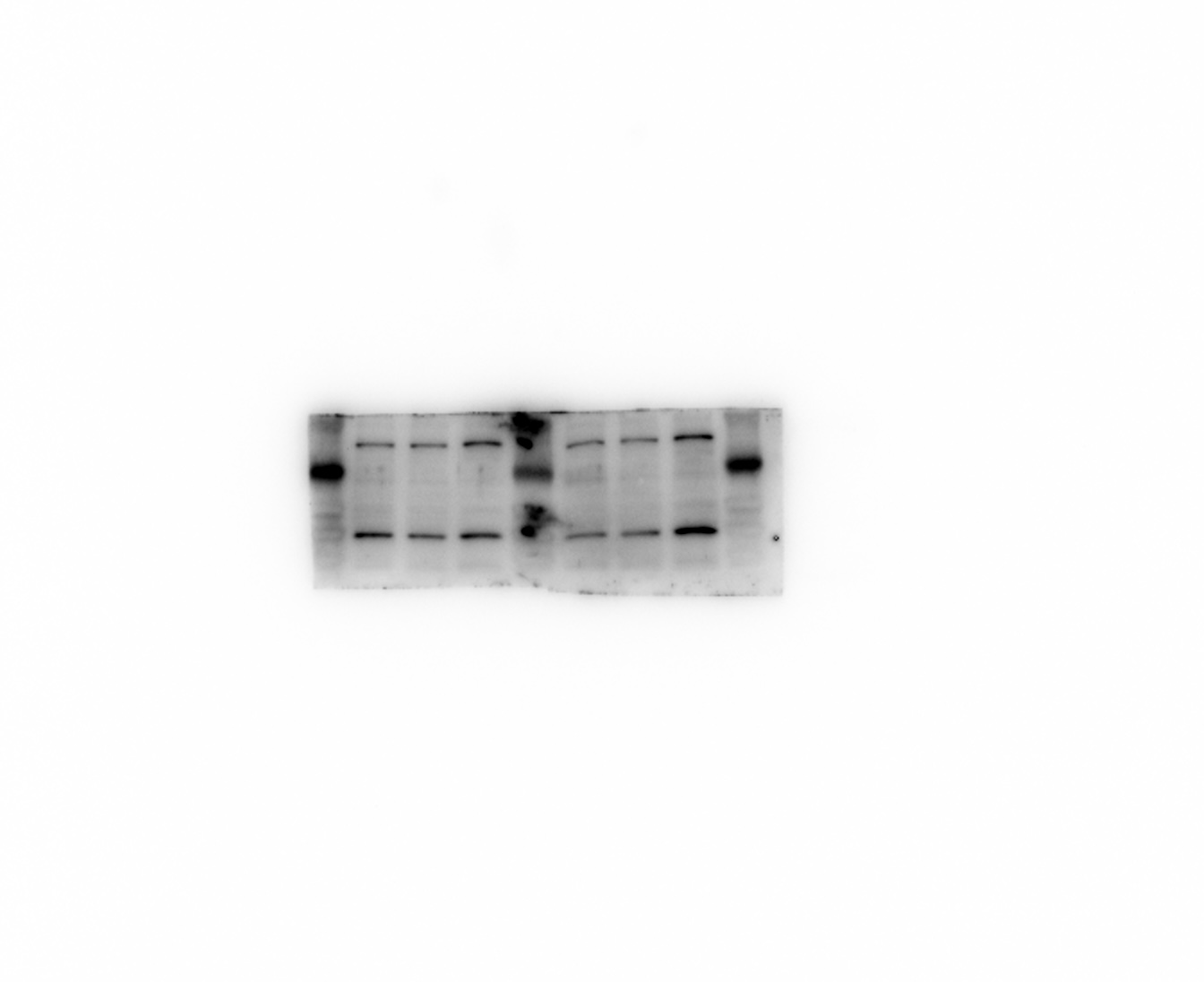



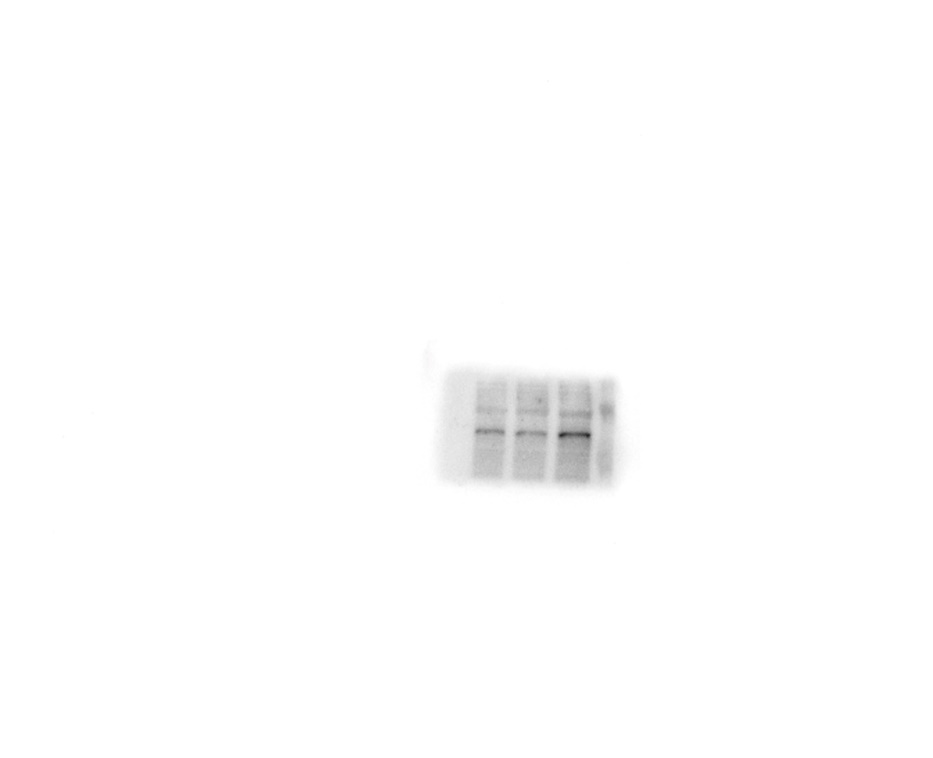

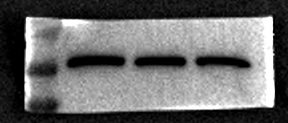


p-ErbB4

150kD

ErbB4

150kD

PPARa

55kD

35kD

PGC1a

100kD

70kD

CPT1A

100kD

70kD

Tubulin

70kD

50kD

**Figure6. A Original blot images**

4weeks

2weeks

ErbB4

**
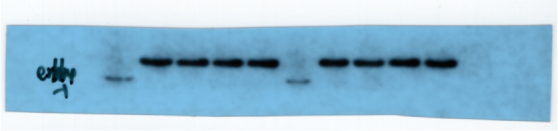
**

4weeks

150kD

2weeks

P-ErbB4

**
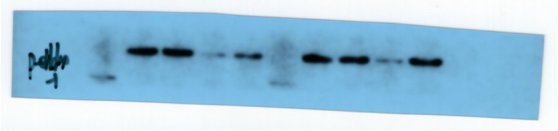
**

150kD

PPARa

**
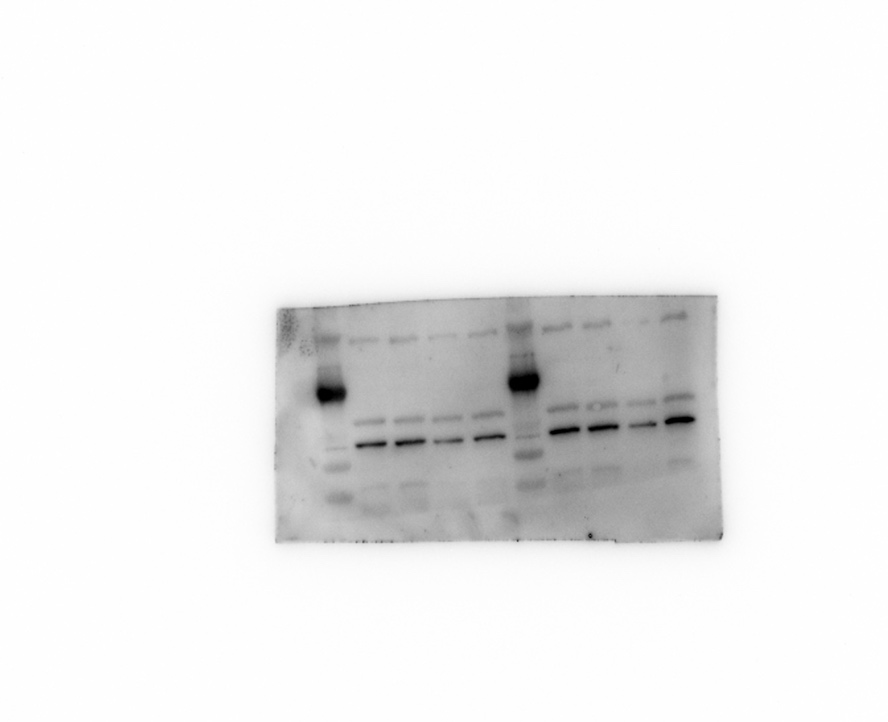
**

50kD

70kD

4weeks

2weeks

PGC1a

PGC1a

**
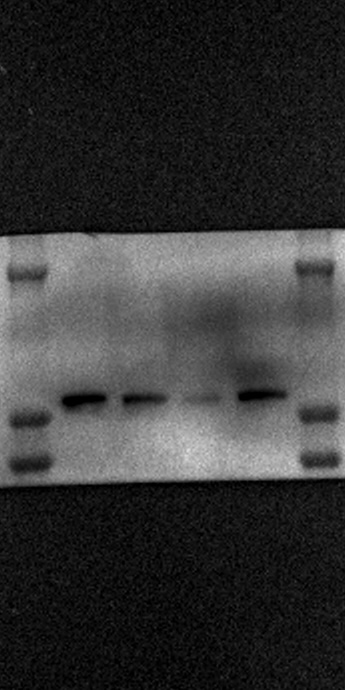

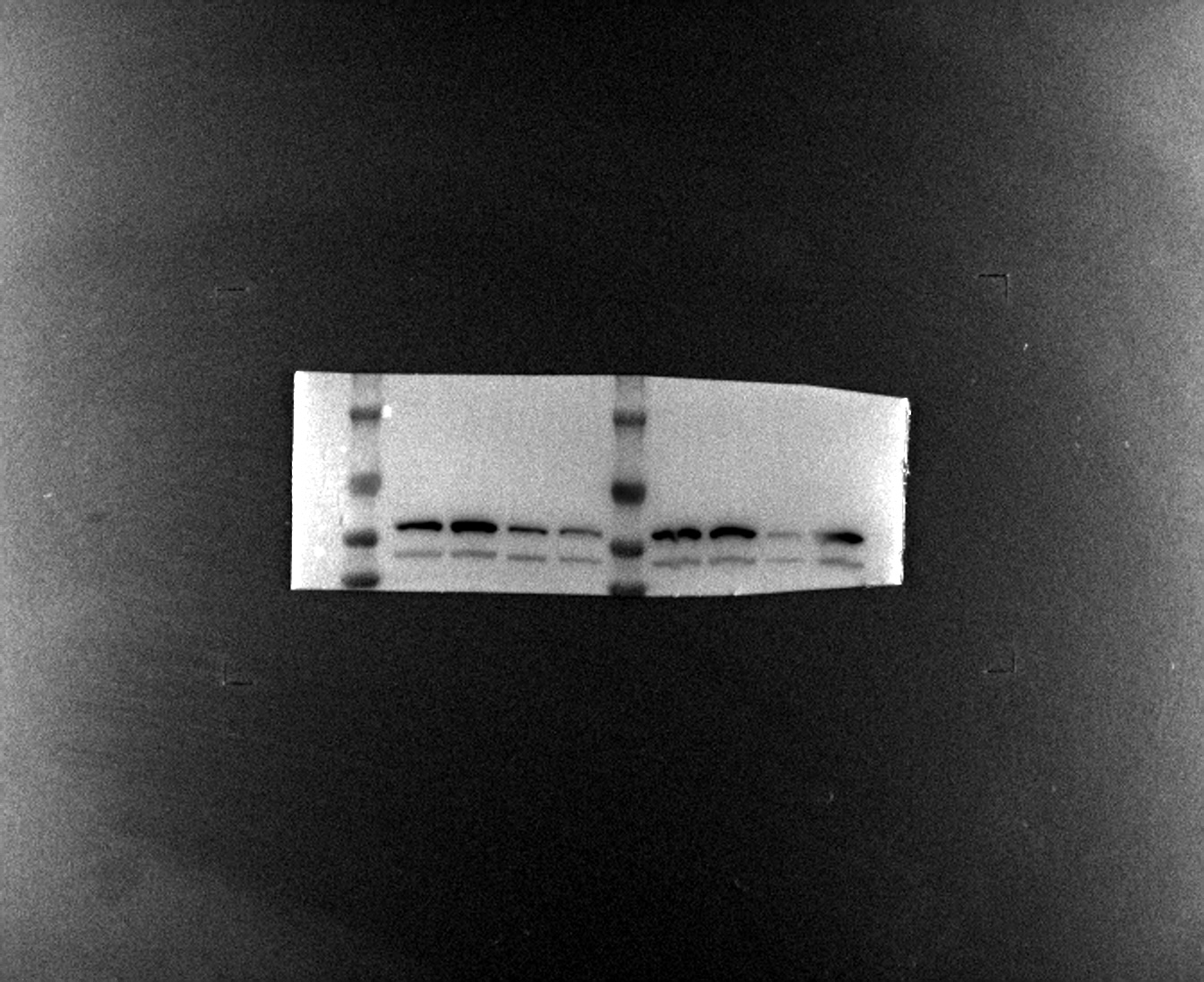
**

67kD

70kD

96kD

100kD

4weeks

2weeks

**
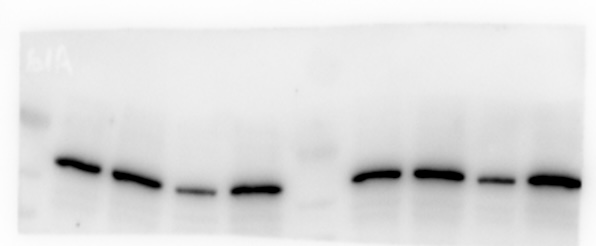
**

70kD

100kD

100kD

70kD

4weeks

2weeks

Tubulin

CPT1A

**
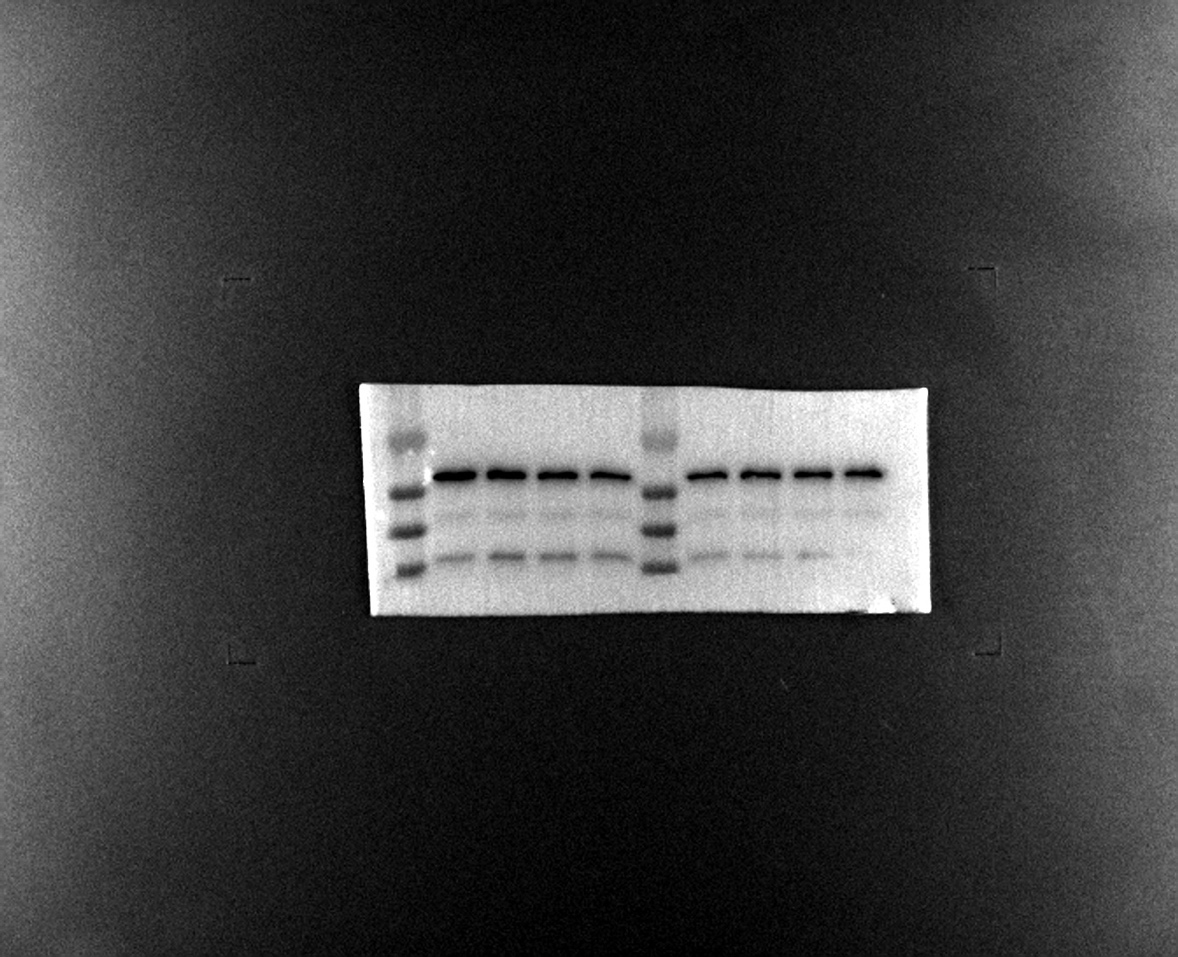
**

50kD

70kD

4weeks

2weeks

**Figure6. B Original blot images**

**
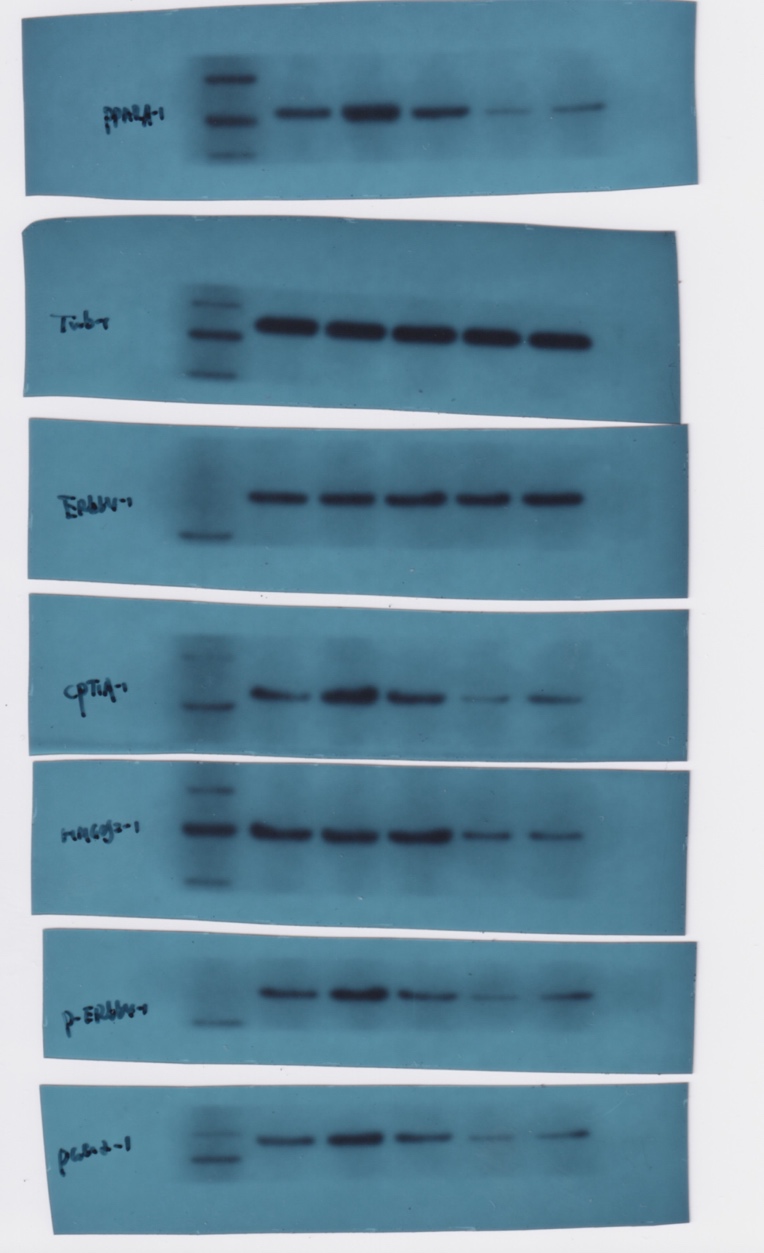
**

50kD

70kD

50kD

70kD

100kD

70kD

70kD

150kD

**
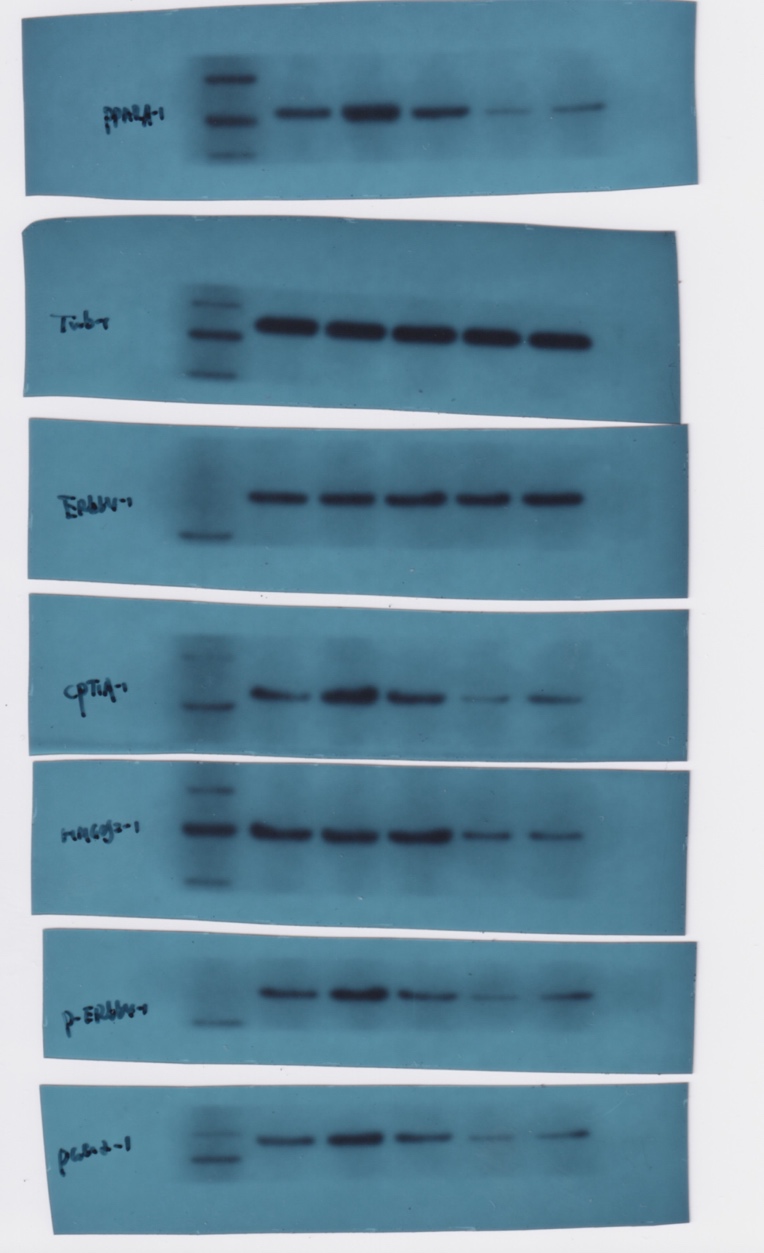
**

100kD

70kD

150kD

**FigureS7A.Original blot images**

Nrg4

**
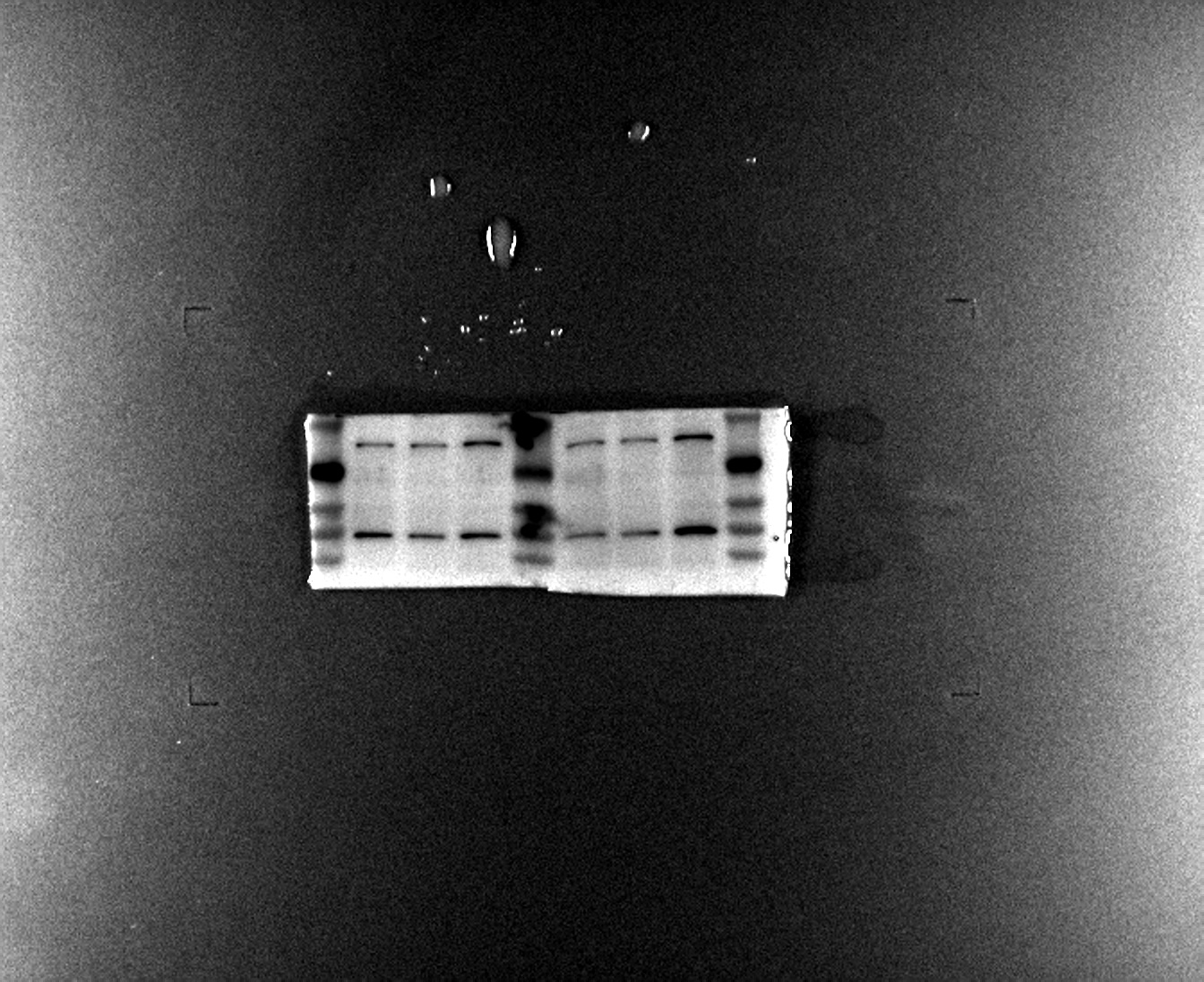
**

70kD

Tubulin

10kD

15kD

25kD

**
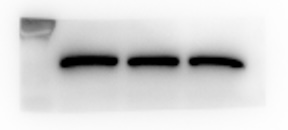
**

50kD

**FigureS7B. Original blot images**


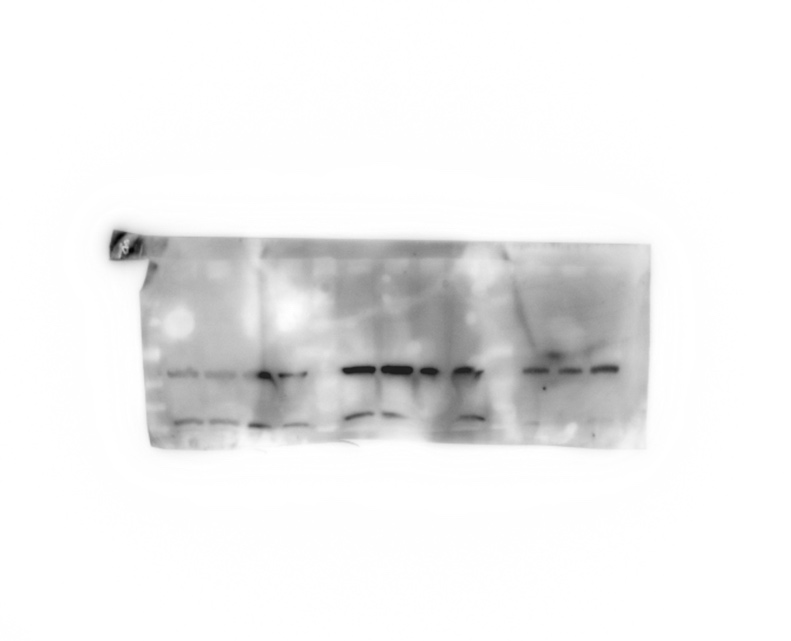


10kD

15kD

Nrg4

Tubulin


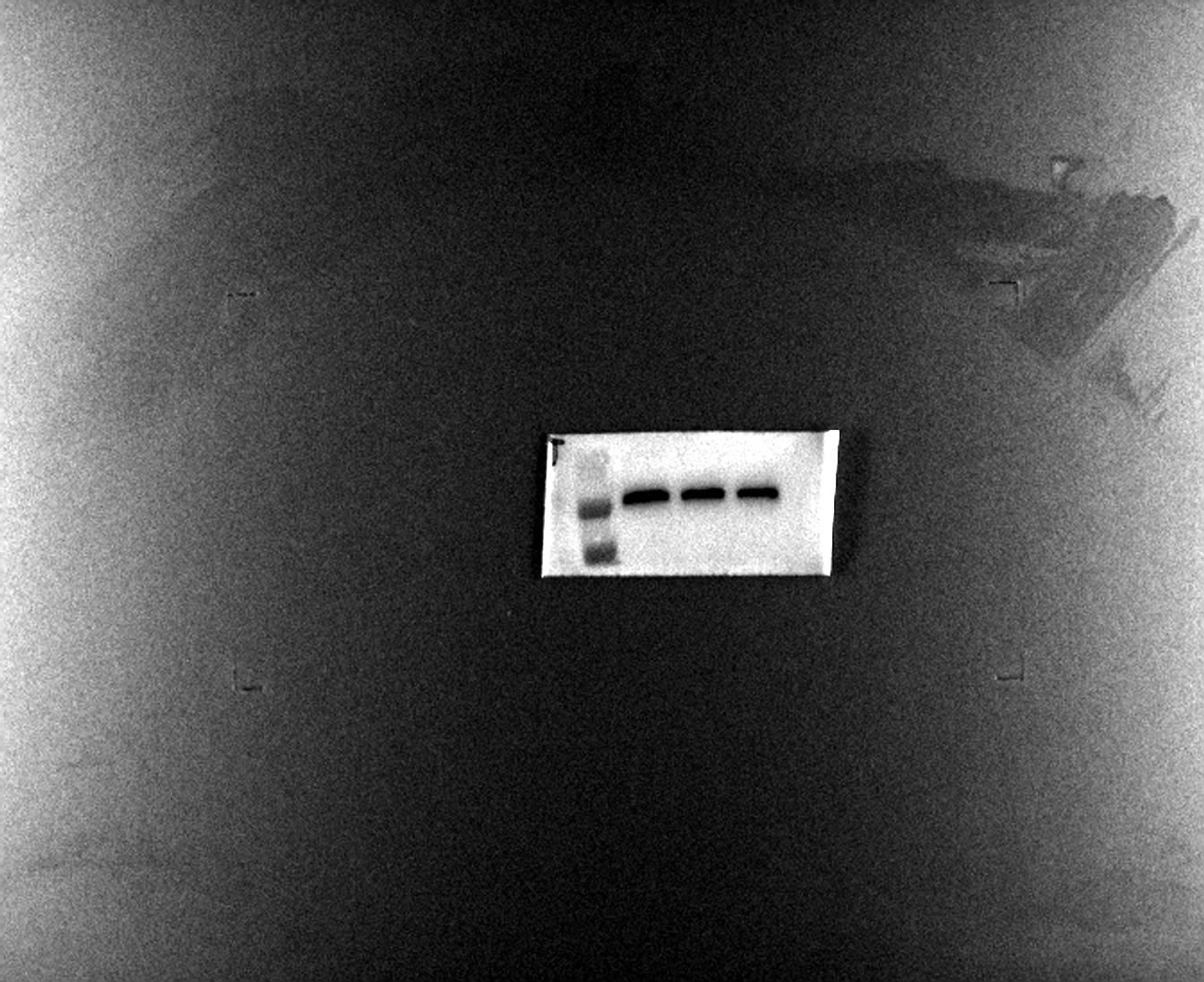


70kD

50kD

**FigureS7F. Original blot images**

ErbB4

**
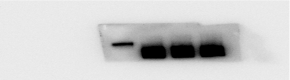
**

250kD

**

**

250kD

p-ErbB4

PPARa

**
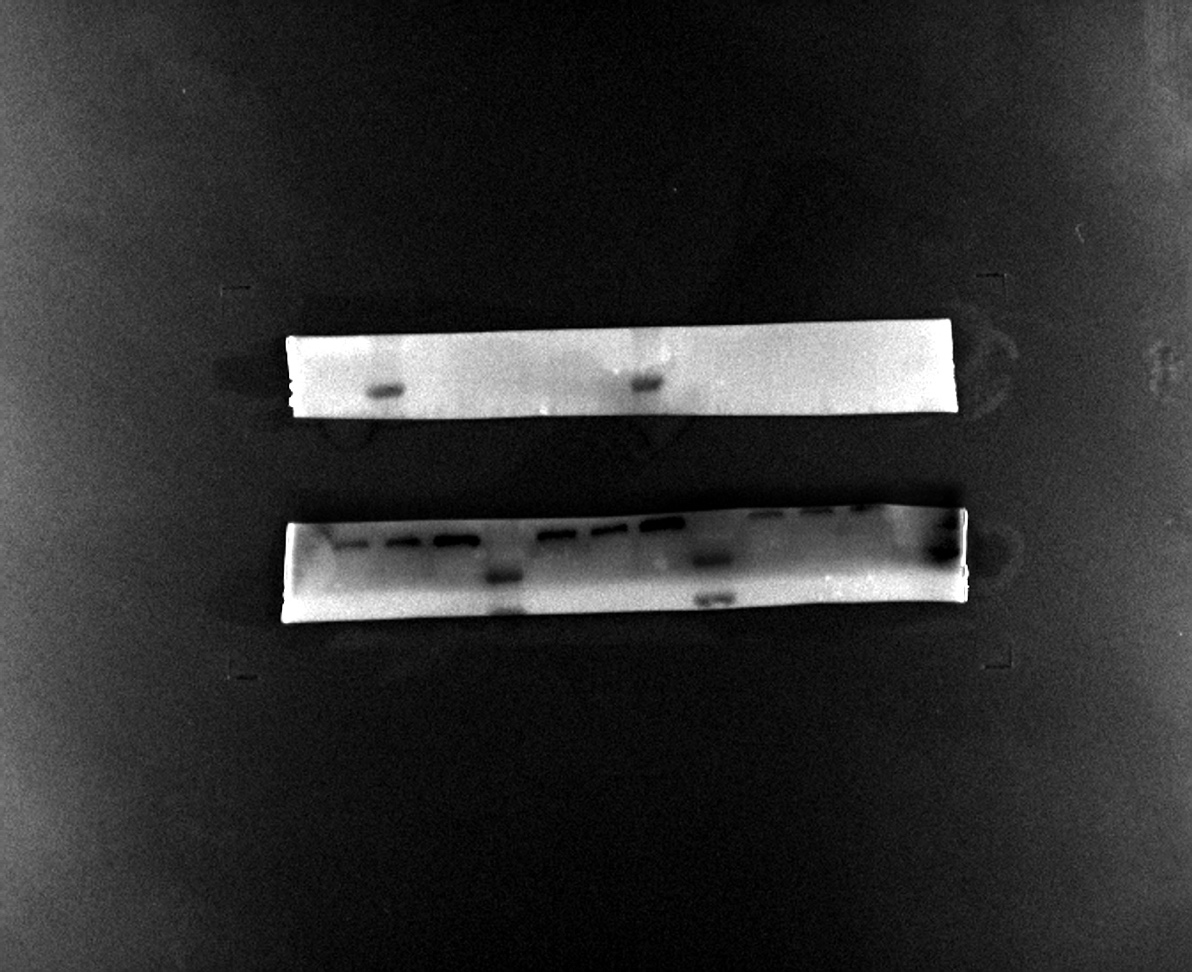
**

50kD

50kD

**
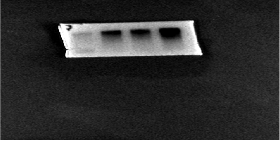
**

70kD

100kD

PGC1a

CPT1A

**
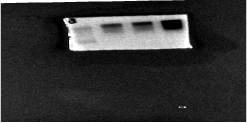
**

100kD

70kD

**
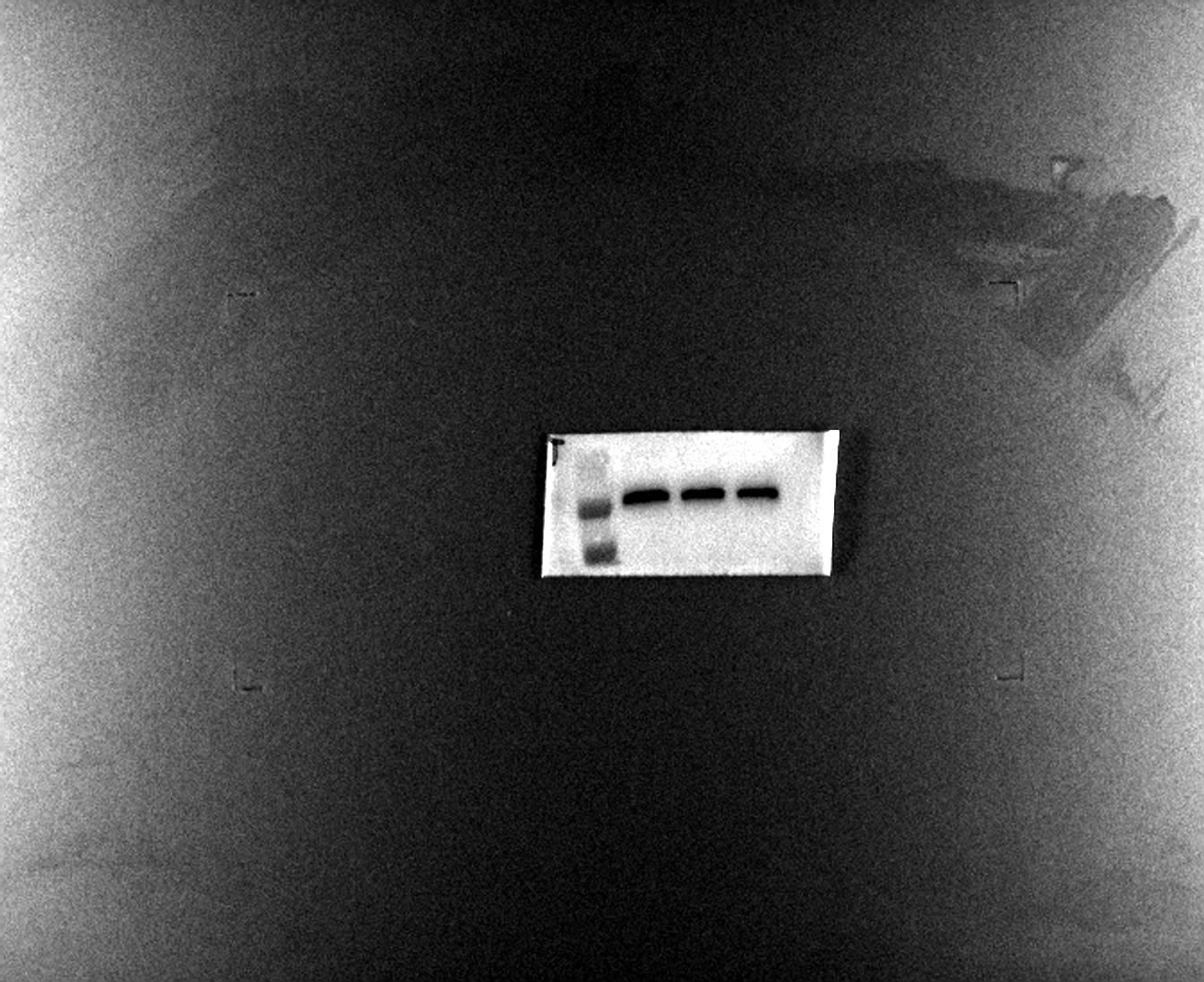
**

50kD

Tubulin
